# Novel high-resolution targeted sequencing of the cervicovaginal microbiome

**DOI:** 10.1186/s12915-021-01204-z

**Published:** 2021-12-16

**Authors:** Karolina M. Andralojc, Mariano A. Molina, Mengjie Qiu, Bram Spruijtenburg, Menno Rasing, Bernard Pater, Martijn A. Huynen, Bas E. Dutilh, Thomas H. A. Ederveen, Duaa Elmelik, Albert G. Siebers, Diede Loopik, Ruud L. M. Bekkers, William P. J. Leenders, Willem J. G. Melchers

**Affiliations:** 1 Department of Medical Microbiology, Radboud University Medical Center, 6500 HB Nijmegen, The Netherlands; 2 Department of Biochemistry, Radboud Institute for Molecular Life Sciences, 6525 GA Nijmegen, The Netherlands; 3 Department of Medical Microbiology, Radboud Institute for Molecular Life Sciences, Radboud University Medical Center, Nijmegen, The Netherlands; 4 Center for Molecular and Biomolecular Informatics, Radboud Institute for Molecular Life Sciences, 6525 GA Nijmegen, The Netherlands; 5 Theoretical Biology and Bioinformatics, Science for Life, Utrecht University, Padualaan 8, 3584 CH Utrecht, The Netherlands; 6 Department of Pathology, Radboud University Medical Center, 6500 HB Nijmegen, The Netherlands; 7 Department of Obstetrics and Gynecology, Radboud University Medical Center, 6500 HB Nijmegen, The Netherlands; 8 Department of Obstetrics and Gynecology, Catharina Hospital, 5602 ZA Eindhoven, The Netherlands; 9 GROW, School for Oncology & Developmental Biology, Maastricht University Medical Centre, 6200 MD Maastricht, The Netherlands; 10 Predica Diagnostics, Toernooiveld 1, 6525 GA Nijmegen, The Netherlands

**Keywords:** Cervicovaginal microbiome, hrHPV, Targeted sequencing, smMIP, CiRNAseq, CST, CIN

## Abstract

**Background:**

The cervicovaginal microbiome (CVM) plays a significant role in women’s cervical health and disease. Microbial alterations at the species level and characteristic community state types (CST) have been associated with acquisition and persistence of high-risk human papillomavirus (hrHPV) infections that may result in progression of cervical lesions to malignancy. Current sequencing methods, especially most commonly used multiplex 16S rRNA gene sequencing, struggle to fully clarify these changes because they generally fail to provide sufficient taxonomic resolution to adequately perform species-level associative studies. To improve CVM species designation, we designed a novel sequencing tool targeting microbes at the species taxonomic rank and examined its potential for profiling the CVM.

**Results:**

We introduce an accessible and practical circular probe-based RNA sequencing (CiRNAseq) technology with the potential to profile and quantify the CVM. In vitro and in silico validations demonstrate that CiRNAseq can distinctively detect species in a mock mixed microbial environment, with the output data reflecting its ability to estimate microbes’ abundance. Moreover, compared to 16S rRNA gene sequencing, CiRNAseq provides equivalent results but with improved sequencing sensitivity. Analyses of a cohort of cervical smears from hrHPV-negative women versus hrHPV-positive women with high-grade cervical intraepithelial neoplasia confirmed known differences in CST occurring in the CVM of women with hrHPV-induced lesions. The technique also revealed variations in microbial diversity and abundance in the CVM of hrHPV-positive women when compared to hrHPV-negative women.

**Conclusions:**

CiRNAseq is a promising tool for studying the interplay between the CVM and hrHPV in cervical carcinogenesis. This technology could provide a better understanding of cervicovaginal CST and microbial species during health and disease, prompting the discovery of biomarkers, additional to hrHPV, that can help detect high-grade cervical lesions.

**Supplementary Information:**

The online version contains supplementary material available at 10.1186/s12915-021-01204-z.

## Background

High-risk human papillomavirus (hrHPV)-induced cervical cancer affects more than half a million women every year [1]. Although the oncogenic role of hrHPV is clear in this process, only a minority of hrHPV infections lead to cervical lesions, and ultimately, cancer. Hence, there is a need to better understand hrHPV-induced alterations in the cervicovaginal environment that contribute to cancer development. Accordingly, recent efforts have focused on the host immune response and the cervicovaginal microbiome (CVM) [2–4]. The latter has a significant role in women’s cervical health and disease [5]. Throughout women’s lives, the CVM can change during the menstrual cycle, pregnancy, or after sexual activities [6–10]. Such microbiome changes also occur in pathogenic conditions like bacterial vaginosis (BV), Candidiasis, and viral infections [11–13]. Interestingly, the composition of the CVM also depends on microbial dominancy and diversity, creating characteristic community state types (CST) that could be either dominant for *Lactobacillus* species (CST I, II, III, and V) or diverse for other bacterial species (CST IV). Variations in the CVM have been widely described in relation to hrHPV infections, with CST IV significantly associated with high-grade cervical lesions and cancer [4, 14, 15]. Furthermore, recent investigations have determined that these microbiome alterations not only occur at the genus level but also at the species level, suggesting that specific microbial species and CST are associated with progressive or regressive behavior of cervical lesions and could act as biomarkers for the disease [16–18]. Nevertheless, studying the CVM and elucidating its function currently relies mostly on short length 16S rRNA gene sequencing (16S rRNA-seq), which struggles to distinguish microbes at this taxonomic rank [19–22].

Microbiome profiling using 16S rRNA-seq is based on the sequence analysis of hypervariable regions (VRs) in ribosomal 16S rRNA genes for microbe identification [23, 24]. PCR amplicons covering two VRs (e.g., V1-V2, V3-V4) are generated with degenerate primer sets and subjected to next-generation sequencing. The technique results in bacterial identification typically providing for family- or genus-level taxonomy, while species identification is achieved for a limited number of genera [25, 26]. Moreover, several studies have observed bias in microbiome profiling with 16S rRNA-seq due to variability in the selection of primers and VRs for amplification and sequencing [27–29]. Since changes in the CVM also take place at the species level, it is essential to develop detection methods with higher resolution and specificity. To this end, Pinna et al. suggest that increasing the number of analyzed VRs may improve the taxonomic resolution in microbiome profiling [30].

Circular probe-based RNA sequencing (CiRNAseq) using single-molecule molecular inversion probes (smMIPs) has proven to be a useful tool for cancer research [31–34] and hrHPV expression studies [35]. smMIPs can be designed to target any nucleic acid sequence and thus could be applied to recognize multiple VRs and to identify diverse microbes such as bacteria, fungi, and viruses simultaneously. Likewise, by targeting and combining multiple VRs for microbiome profiling, CiRNAseq carries a potential to perform high-resolution sequencing with high specificity and sensitivity [30]. Besides being customizable for its targets, the addition of a unique molecule identifier (UMI) to a smMIP corrects for PCR amplification bias, making the counting of amplified smMIPs possible, which could be valuable for absolute microbiome quantification [31, 35]. Because CiRNAseq uses barcode technology, it can handle hundreds of samples in one sequencing run, making the technique cost-effective. Furthermore, it requires fewer specialized skills for data analyses and interpretation than other sequencing methods such as 16S rRNA-seq, making it a handy and accessible technology [36, 37].

We describe here the characteristics and potential of a CVM-specific CiRNAseq assay. We validate the technique’s resolution, specificity, and performance in vitro with mock samples, and profile the CVM of a cohort of cervical smears from women with and without hrHPV-associated cervical abnormalities.

## Methods

### Study participants and samples

For this study, a total of 102 cervical smears in PreservCyt were collected from women participating in the Dutch population-based cervical cancer screening program, which were received and processed at Radboudumc (Nijmegen, the Netherlands). Women participating in the cervical cancer screening program were informed that residual material could be used for anonymous research and had the opportunity to opt-out. Only residual material from women who did not opt-out was included. The histological follow-up outcomes were obtained from the nationwide network and registry of histo- and cytopathology in the Netherlands (PALGA; Houten, the Netherlands). hrHPV identification was performed as previously described [35]. All methods were performed following the institutional guidelines for using human samples. One set of ten hrHPV-positive smears was used for the comparative analyses with 16S rRNA-seq. DNA from these samples was isolated from 1 ml of residual material using DNA and Viral Small volume kit (Roche, cat. no. 6543588001) and subjected to CiRNAseq. The cohort of the remaining 92 cervical smears consisted of 46 hrHPV-positive samples of women with confirmed high-grade cervical intraepithelial neoplasia (CIN2+) and 46 hrHPV DNA-negative smears. Five milliliters of each cervical cell suspension was centrifuged for 5 min at 2500×*g*, and the pellet dissolved in 1 ml of Trizol reagent (Thermo Scientific). RNA was isolated through standard procedures and dissolved in 20 μl nuclease-free water. We routinely processed a maximum of 2 μg of RNA for DNase treatment and cDNA generation, using SuperscriptII (Thermo) as previously described [35].

### smMIP design and targeted sequencing

**Fig. 1:**
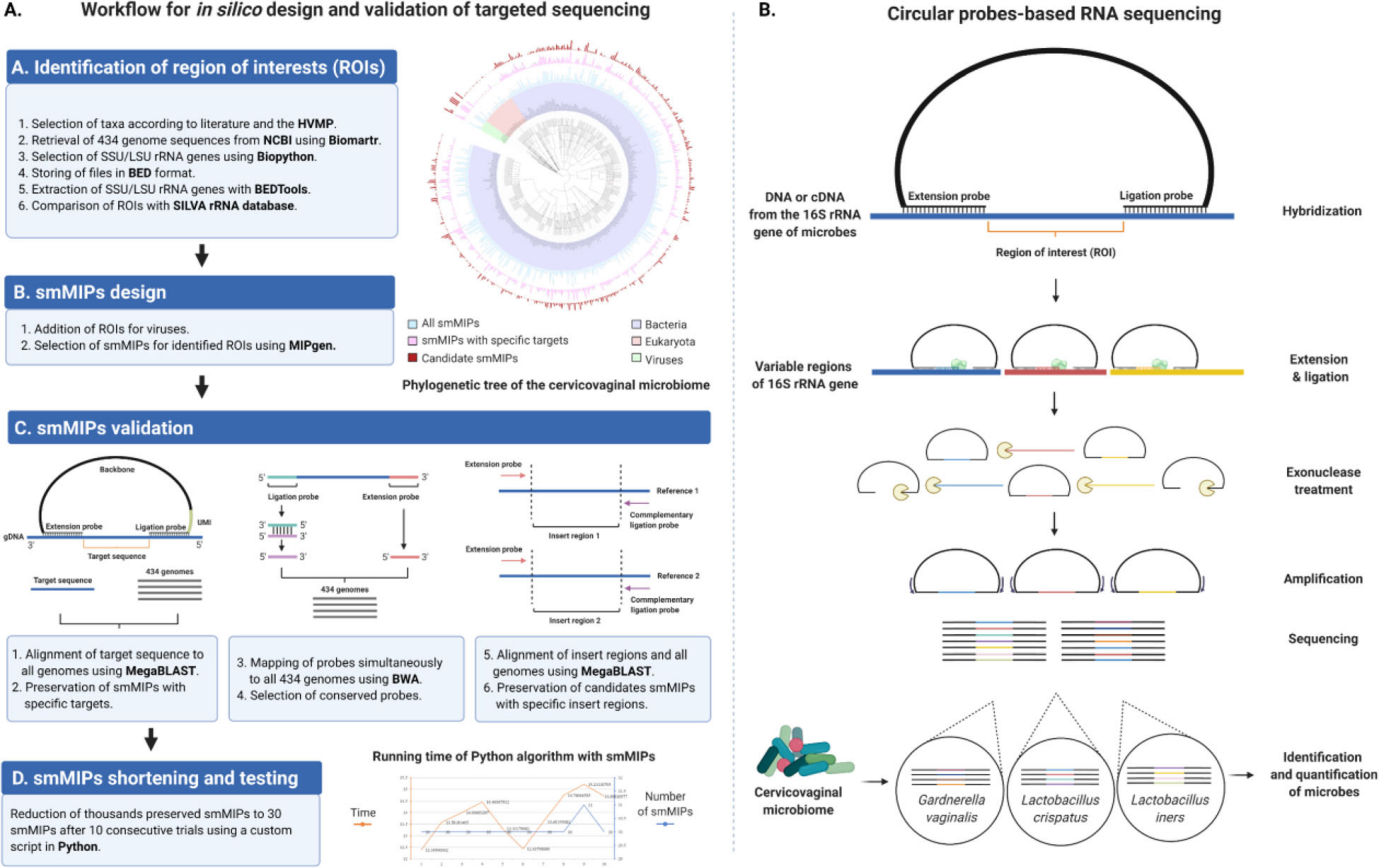
Design and workflow for targeted sequencing of the cervicovaginal microbiome. **A** The design and in silico validation of targeted sequencing involved the initial selection of microbial species from the CVM and their regions of interest (ROIs) within the 16S rRNA gene. Thereafter, single-molecule molecular inversion probes (smMIPs) were designed and validated to target specifically all the ROIs, which resulted in thousands of promising smMIPs. By performing in silico analyses, the amount of smMIPs were shortened and validated to profile all the microbial species, which resulted in the final selection of 30 different smMIPs that are able to achieve high-resolution microbiome profiling. **B** For CVM profiling, these 30 smMIPs are available to hybridize to the 16S rRNA gene of microbes (e.g., DNA or cDNA from RNA) identified as part of the cervicovaginal microbiome. In the cervicovaginal microbiome, hundreds of microbial species can be detected, playing a role in health and disease. smMIPs were selected based on extension and ligation arms that are shared between species and flanking hypervariable ROI that are unique per species. After smMIP hybridization and filling in the ROI gaps, followed by ligation, the library of circularized smMIPs is PCR amplified with barcoded Illumina primers and sequenced. All collected ROI sequences in a sample are then compared to a reference database containing reference ROIs from all microbial species of interest. Based on a combination of two or more ROIs, the microbiome can be annotated in high-resolution. The assay is made quantitative by incorporating a unique molecule identifier (UMI), which eliminated PCR amplification bias

We compiled a list of 434 previously identified microbes that have been recognized as significant in the cervicovaginal environment by recent literature and the Human Vaginal Microbiome Project (Additional file 1) [38, 39]. The genome sequences were initially retrieved from the National Center for Biotechnology Information (NCBI [40]) using Biomartr [41]. Sequences from small ribosomal subunit (SSU) and large ribosomal subunit (LSU) rRNA genes were selected and extracted using Biopython [42] and BEDTools [43], respectively [23, 44]. smMIPs against SSU and LSU rRNA genes were designed in MIPgen [36]. We selected smMIPs with homologous hybridization arms and dissimilar regions of interest (ROIs) and included a random octanucleotide UMI in the smMIP backbone. Next, we compared the selected ROI sequences with the corresponding rRNA sequences within the SILVA rRNA database. Only sequences that were 100% identical over the full length with this database were regarded as fit for annotation [45]. Subsequently, MegaBLAST and the Burrows-Wheeler Aligner (BWA) were combined to validate in silico the specificity of smMIPs in discriminating species [46, 47]. Thereafter, a greedy algorithm was implemented to validate the potential of a smMIP in identifying as many species at once as possible based on ROIs sequences. This validation resulted in the selection of 30 smMIPs targeting the 434 microbes and pathogens (Additional file 2). All smMIPs were validated on a dataset composed of genomes and annotations from species isolated from cervical smears (Fig. 1). Then, to standardize species detection and reduce the chance of false-positive annotation, we considered only species that were identified with two or more reactive smMIPs (on average six probes per species). This filtering resulted in the final selection of our targets consisting of 107 genera and 321 species that represent our cervicovaginal microbiome panel (CVMP), including bacteria, fungi, and parasites (Additional file 3). CiRNAseq was performed as previously described [31, 35]. For the sequencing of individual species, 10 ng of microbial DNA was analyzed. Analyses of cervicovaginal samples were performed on ~ 50 ng of cDNA/DNA generated according to standard protocols (see the “Study participants and samples” section). Following capture hybridization and probe circularization and purification, circularized probes were subjected to PCR with barcoded Illumina primers. After purification of the correct-size amplicons, quality control, and quantification as previously described [35], a 4-nM library was sequenced on the Illumina Nextseq500 platform (Illumina, San Diego, CA) at the Radboudumc sequencing facility.

### CiRNAseq output analysis

Reads were mapped against reference ROIs within our CVMP using the SeqNext module of JSI Sequence Pilot version 4.2.2 build 502 (JSI Medical Systems, Ettenheim, Germany). The settings for read processing were a minimum of 50% matching bases, a maximum of 15% mismatches, and a minimum of 50% consecutive bases without a mismatch between them; for read assigning, the threshold was a minimum of 95% of identical bases with the ROIs. All identical PCR products were reduced to one consensus read (unique read counts, URC) using the UMI. We set an arbitrary threshold of at least 1000 URC from all smMIPs combined in an individual sample, below which we considered an output non-interpretable [48]. For microbial annotation, species with two reactive smMIPs were annotated when 100% of the specific set of smMIPs had URC. Species with three or more reactive smMIPs were annotated when more than 50% of their specific set of smMIPs had URC using a custom R script. For analyses where isolates from our CVMP were not considered, the URC for each isolate were summed to represent the bacterium at the species level. To define relative abundances, microbial species URC was divided by the total URC of all microbes annotated in the sample. For establishing microbial diversity, URC was turned to 1 and 0, indicating the presence or absence of microbes, respectively.

### 16S rRNA gene amplification and sequencing

Residual material from ten hrHPV-positive cervical smears in PreservCyt solution, randomly obtained from the Dutch population-based cervical cancer screening program with approval from the regional institutional review board and the National Institute for Public Health and Environment (No. 2014-1295), was initially pelleted by centrifugation. Pellets were suspended in 1 ml DNA/RNA shield buffer (Zymo, cat. no. R1104). DNA was extracted according to standard protocols and processed by BaseClear B.V. (Leiden, the Netherlands) for microbiome profiling using the primers 357F (5′-CCTACGGGAGGCAGCAG-3′) and 802RV2 (5′-TACNVGGGTATCTAAKCC-3′) that target the V3 and V4 variable regions of the 16S rRNA gene [29]. PCR protocol was as follows: 2 m 95 °C hot start; 35 cycles of 20 s 95 °C, 10 s 61 °C, 15 s 70 °C; 10 m 70 °C. The libraries were barcoded, multiplexed, and sequenced on an Illumina MiSeq machine with paired-end 300 cycles protocol and indexing by BaseClear [49]. Illumina sequencing data were quality checked and demultiplexed by BaseClear standards, and FASTQ files were generated.

### 16S rRNA gene sequencing data analysis

From the FASTQ files, forward and reverse reads were pairwise assembled with PEAR (v0.9.10 [50]) in default settings. For the generation of the 16S-derived taxa-to-sample compositional matrix, a customized Python workflow based on Quantitative Insights Into Microbial Ecology (QIIME v2.0 [51]) was adopted (http://qiime.org). Relative abundances per sample were calculated with QIIME2 default settings, where the reads per taxon were divided by the total number of bacterial reads for that sample.

### In vitro validation of sequencing targets

To test in vitro the specificity and resolution of CiRNAseq, we used 12 bacterial species listed in Additional file 4: Supplementary Table 1, obtained from the Medical Microbiology Department, Radboudumc, Nijmegen, the Netherlands. Bacteria were grown in appropriate culture media. Following growth, their genomic DNA was extracted using DNA and Viral Small volume kit (Roche, cat. no. 6543588001). PCR and Sanger sequencing was performed to validate species identification. Water was used as the negative control. For CiRNAseq, we prepared a concentration of 1.5 ng/μL from each microbes’ DNA in a final volume of 40 μL.

### In silico validation of sequencing microbial species

For testing the specificity of CiRNAseq towards the species *Anaerococcus vaginalis*, *Anaerococcus tetradius*, *Peptostreptococcus anaerobius*, *Gardnerella vaginalis*, *Bifidobacterium longum*, and *Prevotella buccalis* in silico, we downloaded the 16S rRNA gene of the species *A. vaginalis* (D14146), *A. tetradius* (D14142), *P. anaerobius* (D14150), *G. vaginalis* (M58744), *Bifidobacterium longum sp. suillum* (AB924532), and *P. buccalis* (AB547676) from the LPSN database (DSMZ, Germany). SnapGene® Viewer 5.3.2 (Insightful Science; snapgene.com) was used to verify the hybridization of the ROIs that are targeted by the smMIPs to detect the species and the hybridization of the primers 357F and 802RV2 to the V3-V4 regions*.* Clustal Omega v1.2.4 [52] was used to align ROIs sequences from each compared species. Pairwise sequence similarities and phylogenetic analyses were calculated using the method recommended by Meier-Kolthoff et al. [53] for the 16S rRNA gene via the GGDC web server [54] available at https://ggdc.dsmz.de/

### In vitro validation of RNA testing and quantification

To assess the capacity of CiRNAseq to quantify and analyze microbial RNA, an *Escherichia coli* (*E. coli*; ATCC 25922) culture in stationary phase was inoculated at 5% in BHI medium and incubated at 37 °C on a shaking platform at 100 rpm for 48 h. Optical density (OD630) was measured every hour, and 1-ml aliquots were taken after each measurement, pelleted, and stored for nucleic acid isolation. After 26.5 h of culture, an aliquot was taken for autoclaving. A second aliquot was treated with 0.75 ml of cefoxitin (1 mg/ml), followed by further growth for an additional 20 h (Additional file 5: Supplementary Table 2). Nucleic acids were isolated from all aliquots using the MagNA Pure kit (Roche, cat. no. 03730964001). RNA concentrations (ng/ml) were measured using NanoDrop 2000 (Thermo Scientific). After treatment with DNAase, RNA was processed to cDNA for CiRNAseq analysis.

### Statistical analyses

Analyses with our CVMP were performed using ClustVis [55]. For the clustering analysis (Fig. 5A), the settings were as follows: clustering distance for columns: Canberra [56, 57]; clustering method: Ward (unsquared distances); row scaling: Pareto scaling [58]. Canberra distance normalizes the absolute difference in abundance of each taxon, allowing comparison of minor taxa. A shorter Canberra distance indicates greater similarity.

Linear discriminant analysis (LDA) effect size was performed using the LEfSe tool [59]. LEfSe combines standard tests for statistical significance (Kruskal-Wallis test and pairwise Wilcoxon test) with LDA for feature selection. Alpha value for the factorial Kruskal-Wallis test was 0.05. Threshold on the logarithmic LDA score for discriminative features was 2.0 [59].

Microsoft Excel 2016® and GraphPad Prism v9.0.0 (GraphPad Software, Inc., USA) were used to analyze datasets and determine species richness, Shannon’s diversity index, and Pearson’s r correlations. The statistical significance of differences in microbial richness, diversity, and relative abundance was calculated using GraphPad with the Mann-Whitney test to obtain the *p*-value. Significant differences between groups are denoted by * *p* < 0.05, ** *p* < 0.01, *** *p* < 0.001, or **** *p* < 0.0001.

## Results

CiRNAseq is a sequencing tool for high-resolution microbiome profiling that uses smMIPs to target multiple VRs of the 16S rRNA gene characterizing the composition, abundance, and diversity of the CVM. Following the identification of relevant microbes in the cervicovaginal niche and their regions of interest (ROIs) (Fig. 1A), we designed probes with homologous hybridization arms with high specificity for ribosomal RNA, that flank heterologous ROIs (Fig. 1A). With bioinformatic analyses (see the “Methods” section), we validated thousands of smMIPs to select only 30 that combined can detect 107 genera and 321 species within the CVM (Fig. 1A). The selected probes were subjected to an in silico validation to ensure accurate microbiome profiling (Fig. 1A) [38, 39]. By comparing these ROIs with a reference database, this method assigns URC to microbes of interest. Because we require that at least two different ROIs must be detected in a microbe, the CiRNAseq pipeline ensures a robust species-level annotation of the microbiome (Fig. 1B).

We performed in vitro and in silico validations to demonstrate the potential of CiRNAseq for high throughput sequencing of the microbiome and compared this new method to 16S rRNA-seq. We designed a dedicated CiRNAseq test to study the CVM in smears from hrHPV-negative women and women with hrHPV-associated CIN2+ lesions. We also validated the specificity, resolution, reproducibility, targeting (DNA/RNA), and quantification abilities of the technology in profiling the CVM.

### CiRNAseq exhibits high specificity and resolution

**Fig. 2:**
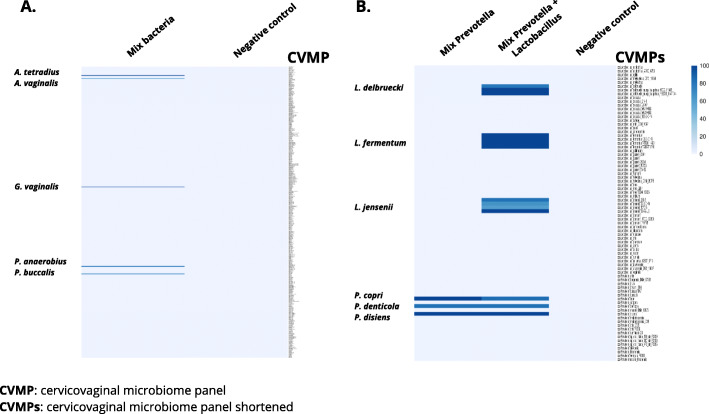
CiRNAseq exhibits high specificity and resolution. **A** CiRNAseq exhibits high specificity in a mixed microbial sample. The method can discriminate different microbes in a single sample of mixed bacteria. **B** CiRNAseq displays high-resolution in detecting microbes. The technique can identify different species of the same genus such as *P. copri*, *P. denticola*, and *P. disiens* and other species from a distinctive genus such as *L. delbruecki*, *L. fermentum*, and *L. jensenii* within the same sample. The CVMP was shortened (CVMPs) in **B** to only display species and isolates from *Lactobacillus* and *Prevotella* genera. Values represent the percentage of reactive smMIPs in the specific set for each microbe. Negative control: water

To validate the specificity of CiRNAseq in a mixed microbial environment, we first tested the technique by analyzing a defined mixture of genomic DNA from *Anaerococcus tetradius*, *Anaerococcus vaginalis*, *Gardnerella vaginalis*, *Peptostreptococcus anaerobius*, and *Prevotella buccalis*, which are typical for the CVM (Fig. 2A, Additional file 4: Supplementary Table 1, Additional files 7 and 8). Water was used as a negative control. CiRNAseq correctly identified the five input species based on sequence comparison with the reference ROIs. In the negative control, the technique did not yield any reads (Fig. 2A). In silico analyses using the 16S rRNA gene of these exact species and their ROIs (Additional file 6: Supplementary Figure 1, and Additional file 9) further confirm that the strict technique’s requirements for species annotation (see the “Methods” section) facilitate an accurate discrimination of microbes in a mixed microbial sample with high specificity.

Subsequently, we assessed the technique’s resolution in detecting microbes at the species level (Fig. 2B, Additional file 4: Supplementary Tables 1, 7 and Additional file 10). To this end, we prepared a mixed microbial sample consisting of genomic DNA from three species of *Prevotella* (*Prevotella copri*, *Prevotella denticola*, and *Prevotella disiens*) and added these to a second mixed sample containing DNA from three *Lactobacillus* species (*Lactobacillus delbruecki*, *Lactobacillus fermentum*, and *Lactobacillus jensenii*). All of these species are commonly found in the CVM. As represented in Fig. 2B, CiRNAseq correctly identified all individual species in all samples. Thus, CiRNAseq is able to distinguish microbes at the species level for this specific mixed microbial sample, showing potential for high-resolution sequencing of the CVM.

### CiRNAseq RNA quantification capacity mirrors bacterial growth and activity

**Fig. 3:**
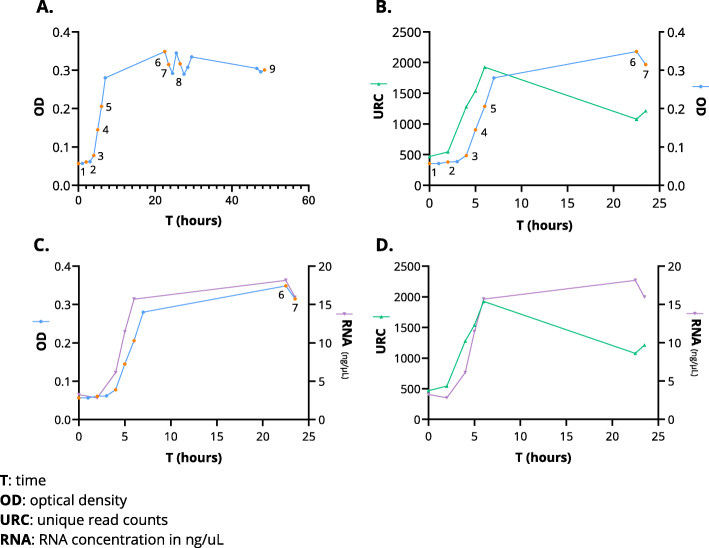
CiRNAseq RNA quantification capacity mirrors bacterial growth and activity. **A** The OD obtained from monitoring *E. coli* growth for 48 h reveals the bacterial growth phases. The nine orange time points indicate the phases from when samples were taken for sequencing analyses. **B** *E. coli* URC correlated with the OD, particularly from the lag to the exponential phases. Samples taken in the stationary phase had lower URC than the last measurement within the exponential phase. **C** RNA concentrations of the samples taken for sequencing are parallel to the OD, indicating that low URC found in time points six and seven may reflect the measurement of ribosomal activity. **D** RNA concentrations also match the URC obtained from sequencing

In natural niches such as the CVM, DNA is a very stable molecule, while RNA is rapidly degraded. Therefore, whereas DNA sequencing can reveal the presence of genomic DNA of bacterial species in a sample, RNA sequencing gives information on the activity of such species by identifying which genomic regions are transcribed to RNA [60]. To evaluate the CiRNAseq potential in quantifying active microbes at the RNA level, we examined how the growth of *E. coli*, a species that can be found in the CVM [61, 62], is reflected in the number of unique read counts (URC) obtained from RNA sequencing. Following the growth of a pure culture of *E. coli* for 48 h through OD measurement every hour, we selected nine-time points where the *E. coli* culture was sampled for RNA isolation, including the bacterial lag, exponential, and stationary phases (Fig. 3A, in orange dots). We also selected two samples that were either autoclaved or treated with an antibiotic (Additional file 5: Supplementary Table 2). Samples were taken in duplicate and subjected to CiRNAseq to test reproducibility (Additional file 11: Supplementary Figure 2A). The mean number of URC achieved in these replicates for the lag and exponential phases is shown in Fig. 3B (green line, first seven-time points) and Additional file 11: Supplementary Figure 2B. When comparing the OD of *E. coli* culture to the mean of URC obtained from sequencing, we found that the values were significantly correlated, particularly from the lag to the exponential phase (*p* = 0.0286) (Fig. 3B). Samples taken from the stationary growth phase had lower URCs, indicating lower ribosomal activity in bacteria from the stationary phase than bacteria from the exponential growth phase.

We also analyzed the RNA concentrations of each aliquot taken for sequencing and compared them to the OD and URC, as shown in Fig. 3C, D, and Additional file 11: Supplementary Figure 2C. Here we noticed that the isolated total RNA matched the OD of *E. coli* growth phases (Fig. 3C). Furthermore, we observed that the RNA levels of the samples taken from the stationary phase (time points six and seven) were higher than those from the exponential phase (Fig. 3D), reflecting the accessible RNA for sequencing. As expected, we did not find any URC after autoclaving the sample taken in time point eight, even though the OD and RNA concentration measured previous to autoclavation was similar to the growth phase (Additional file 11: Supplementary Figure 2C). Similarly, the sample treated with cefoxitin (antibiotic) had a low number of URC, suggesting inhibition of ribosomal activities (Additional file 11: Supplementary Figure 2C). Thus, CiRNAseq can quantify microbes’ RNA, mirroring translational activity and growth.

### CiRNAseq provides genus-level microbiome profiling as 16S rRNA-seq but offers improved taxonomic resolution

Given that the gold-standard sequencing method for profiling the microbiome is 16S rRNA-seq, we compared both sequencing methodologies. First, we did an in silico comparison analyses between both techniques based on the 16S rRNA sequences of two different species within the family *Bifidobacteriaceae*. Species from the genera *Gardnerella* and *Bifidobacterium* can be found in the CVM and studies have described that 16S rRNA-seq struggles differentiating their species due to limited variability of the used VRs for microbiome profiling [8, 28, 29, 63]. As shown in Additional file 12: Supplementary Figure 3, the V3/4 regions of the species *Bifidobacterium longum* and *Gardnerella vaginalis* have > 90% similarity. Our designed smMIPs also target V5, V6, and V9 regions that have < 45% homology and therefore can discriminate the species with higher confidence (Additional file 5).

**Fig. 4:**
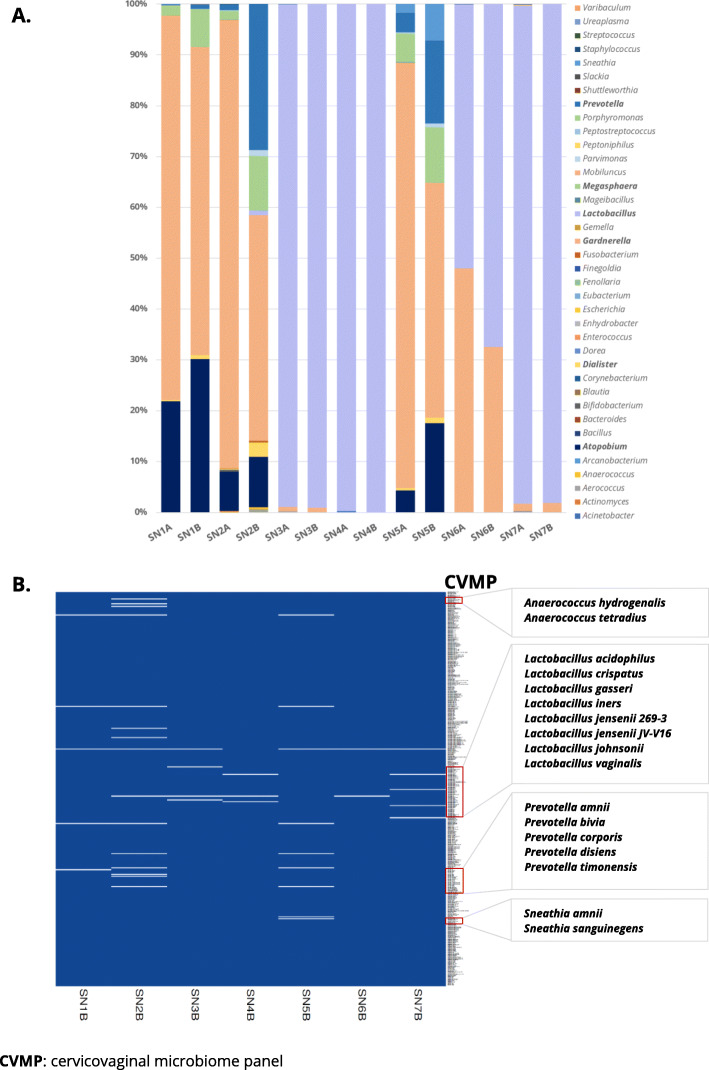
CiRNAseq provides genus-level microbiome profiling as 16S rRNA-seq but offers improved taxonomic resolution. **A** 16S rRNA-seq (SN-A) and CiRNAseq (SN-B) possess similar sequencing capacity when differentiating 31 of the 38 genera analyzed. The methods gave the same results with respect to quantifying the genera *Lactobacillus*, *Gardnerella*, *Atopobium*, and *Megasphaera*. The microbial composition of samples A and B is similar when analyzed using the two sequencing techniques. Microbial species and isolates URC were summed to show the results at the genus level. **B** CiRNAseq allows detecting bacteria at high-resolution. The technique suggested 24 different bacterial species, including two species of *Anaerococcus*, seven *Lactobacillus* specie*s*, five species of *Prevotella*, and two species of *Sneathia*. For **A**, values represent the relative abundances of each microbe in the sample. Bacterial species isolates within our CVMP were considered for display of **B**

Next, we randomly selected ten hrHPV-positive smears, which were simultaneously profiled using CiRNAseq and 16S rRNA-seq at the DNA level. Two out of ten samples had low reads (< 2500 reads) with 16S rRNA-seq compared to the rest of the samples (> 80,000 reads) and were excluded from the analyses. One additional sample had < 1000 URC with CiRNAseq and was also excluded from the study. In the remaining seven samples, we determined the relative microbes’ abundances. Following 16S rRNA-seq, we focused our analyses on 38 genera that were profiled by 16S rRNA-seq and were also available for microbiome profiling using CiRNAseq (Fig. 4 and Additional files 3, 13 – 15). Microbes with relative abundances ≤ 0.07% were considered non-present in the samples.

The seven remaining samples sequenced with 16S rRNA-seq (SN-A) and CiRNAseq (SN-B) were analyzed, as shown in Fig. 4. Here, we first observed that the relative abundances are highly similar using both techniques (Fig. 4A), suggesting that CiRNAseq and 16S rRNA-seq have a comparable efficiency in microbial identification and quantification. This finding can be easily observed in samples 3, 4, 6, and 7 (A and B), where both techniques detected *Lactobacillus* with equivalent relative abundances (*r* = 0.9605, *p* = 0.0006, Fig. 4A and Additional file 16: Supplementary Figure 4A). Likewise, both methods yielded similar relative abundances for *Gardnerella* (*r* = 0.9384, *p* = 0.0018, Fig. 4A and Additional file 16: Supplementary Figure 4B), *Atopobium* (*r* = 0.9255, *p* = 0.0028, Fig. 4A and Additional file 16: Supplementary Figure 4C), and *Megasphaera* (*r* = 0.8344, *p* = 0.0196, Fig. 4A and Additional file 16: Supplementary Figure 4D). Still, 16S rRNA-seq yielded a lower relative abundance than CiRNAseq for the genera *Dialister*, *Parvimonas*, *Prevotella*, and *Sneathia* (Fig. 4A).

Both techniques profiled *Gardnerella*, *Atopobium*, *Aerococcus*, *Dialister*, *Lactobacillus*, *Megasphaera*, *Parvimonas*, *Prevotella*, and *Sneathia*. CiRNAseq also detected *Anaerococcus* and *Fusobacterium* in higher relative abundances than 16S rRNA-seq (Fig. 4A). Genera *Actinomyces*, *Bifidobacterium*, *Corynebacterium*, *Peptoniphilus*, and *Ureaplasma* were detected by 16S rRNA-seq (relative abundances between 0.10 and 0.40%), but not by CiRNAseq (Fig. 4A). From the 24 genera that 16S rRNA-seq yielded ≤ 0.07% in relative abundances, CiRNAseq was concordant in 22 (91%). In general, 16S rRNA-seq and CiRNAseq were concordant in 31 out of the 38 genera analyzed (81%), illustrating the technique’s specificity and sensitivity at the genus level.

To further investigate the species resolution of CiRNAseq in the CVM, we also analyzed samples SN1B to SN7B at this taxonomy level, as shown in Fig. 4B, Table 1, and Additional file 17. In total, we observed 24 different species from our CVMP. We were able to detect two species of *Anaerococcus*, seven species of *Lactobacillus*, five species of *Prevotella*, and two species of *Sneathia* (Fig. 4B, Table 1). When considering the classification of cervicovaginal CST [5], CiRNAseq also allows the characterization of CST IV (SN1B, SN2B, and SN5B; high diversity), CST III (SN3B and SN6B; *L. iners* dominance), and CST I (SN4B and SN7B; *L. crispatus* dominance), which also resembled the composition defined by 16S rRNA-seq (Fig. 4A, B). Therefore, these CiRNAseq results suggest the ability to identifying bacteria at the species level and in microbial communities with high specificity in the complex CVM niche.

**Table 1:** Species-level identification using circular probe-based RNA sequencing

Bacterial species	SN1B	SN2B	SN3B	SN4B	SN5B	SN6B	SN7B
*Aerococcus christensenii*		•					
*Anaerococcus hydrogenalis*		•					
*Anaerococcus tetradius*		•					
*Atopobium vaginae*	•	•			•		
*Dialister micraerophilus*	•	•			•		
*Fenollaria massiliensis*		•					
*Fusobacterium nucleatum*		•					
*Gardnerella vaginalis*	•	•	•		•	•	•
*Lactobacillus acidophilus*			•				
*Lactobacillus crispatus*				•			•
*Lactobacillus gasseri*							•
*Lactobacillus iners*		•	•	•		•	
*Lactobacillus jensenii*			•	•			
*Lactobacillus johnsonii*							•
*Lactobacillus vaginalis*							•
*Megasphaera genomosp type 1*	•	•			•		
*Parvimonas micra*		•			•		
*Prevotella amnii*		•			•		
*Prevotella bivia*	•						
*Prevotella corporis*		•					
*Prevotella disiens*		•					
*Prevotella timonensis*		•			•		
*Sneathia amnii*					•		
*Sneathia sanguinegens*					•		

### CiRNAseq: CVM changes in women with hrHPV-induced lesions

Several studies suggest that accurate detection of microbial species in the CVM may be relevant for predicting the progression of hrHPV-induced precancerous cervical lesions and cancer [15, 64–66]. To investigate this, we applied CiRNAseq to RNA isolated from cervical smears of hrHPV-negative women (considered healthy, *n* = 46) and women with hrHPV-positive high-grade cervical intraepithelial neoplasia (CIN2+, *n* = 46) (Additional files 18 and 19).

**Fig. 5:**
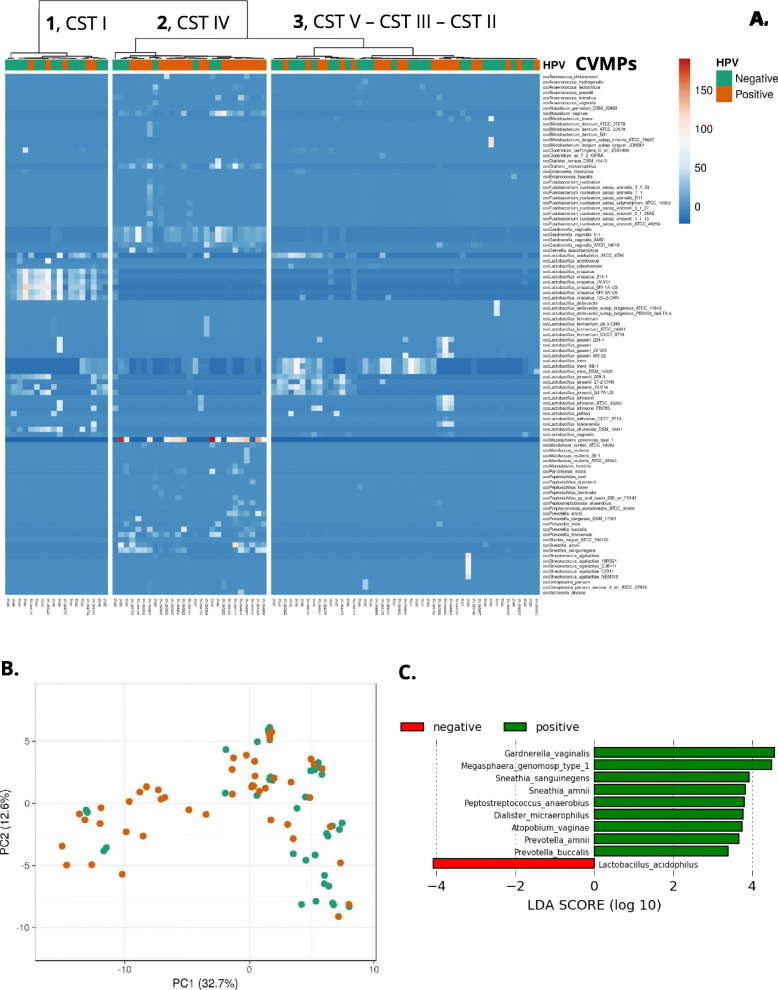
CiRNAseq: the CVM changes upon hrHPV infection. **A** Unsupervised clustering analysis of randomly selected cervical smears from hrHPV-negative and hrHPV-positive women (CIN2+) profiled at the RNA level shows three distinct clusters from left to right: the first cluster (*1*) includes a higher proportion of hrHPV-negative women, who have a microbiome characterized of *Lactobacillus* species, and particularly *L. crispatus* (CST I). The second cluster (*2*) contains a higher proportion of hrHPV-positive women with CIN2+ lesions, who possess a diverse microbiome (CST IV) containing distinctive bacteria such as *Atopobium vaginae*, *Dialister micraerophilus*, *Gardnerella vaginalis*, *Lactobacillus iners*, *Megasphaera genomosp type 1*, *Sneathia amnii*, and *Sneathia sanguinegens*. The third cluster (*3*) includes both hrHPV-negative and hrHPV-positive women with predominantly hrHPV-negative women, who have a unique microbiome characterized by *Lactobacillus* species such as *L. gasseri* (CST II), *L. iners* (CST III), *L. jensenii* (CST V), and *L. acidophilus*. Clustering distance for columns: Canberra; clustering method: Ward (unsquared distances); Row scaling: Pareto scaling. The CVMP was shortened (CVMPs) to only include species with URC. URC from bacterial isolates in our CVMP were considered for analysis. **B** Principal component analysis (PCA) shows that hrHPV-negative and hrHPV-positive samples are correlated with PC1. The loading score of PC1 (data not shown) indicates that anaerobic bacteria have the stronger association with PC1 (Additional file 19). Original values are ln(x + 1)-transformed. No scaling is applied to rows; SVD with imputation is used to calculate principal components. **C** Histogram of the LDA scores computed for features differentially abundant between hrHPV-negative (negative) and hrHPV-positive women (positive)

Unsupervised clustering analysis using URC from each microbial species in individual samples of our cohort is shown to generate three clusters (Fig. 5A). The clusters represented the well-known community state types (CST) [5]. Cluster 1 consisted of 18 samples, of which 72.2% were hrHPV negative, and was characterized by a CST I that is dominated by *L. crispatus*. Additional *Lactobacillus* specie*s* such as *L. iners*, *L. jensenii*, *L. ultunensis*, and *L. acidophilus* were also common (Fig. 5A). With a Fisher’s exact test, CST I showed a small association to hrHPV-negative women (*p* = 0.0639) when compared to hrHPV-positive women in cluster 1.

Cluster 2 consisted of 27 samples, of which 20 (74%) were from women with hrHPV-induced high-grade lesions. These women had a CVM consistent with CST IV, characterized by depletion of *Lactobacillus* species and colonization of mainly anaerobic bacteria such as *M. genomosp type 1*, *G. vaginalis*, *S. amnii*, *S. sanguinegens*, *D. micraerophilus*, and *A. vaginae*. With a Fisher’s exact test, CST IV exhibited a significant association to hrHPV-positive women (*p* = 0.0055) when compared to hrHPV-negative women in cluster 2. The third cluster (3) contained 47 samples, of which 26 (55.3%) were hrHPV negative and 21 (44.7%) had hrHPV-induced lesions. Women’s CVM in cluster 3 were still dominant for *Lactobacillus* specie*s*, and their microbial composition was consistent with other CST such as II (dominance for *L. gasseri*), III (dominance for *L. iners*), and V (dominance for *L. jensenii*) (Fig. 5A).

We also tested our cohort of 92 samples through a principal component analysis (PCA). We determined PC1 and PC2, representing 32.7% and 12.6% of our cohort, respectively (Fig. 5B). Here, we observed a minor separation of samples corresponding to both hrHPV-negative and hrHPV-positive women with some overlap. After analyzing the loading score of PC1 (Additional file 20), we found that anaerobic bacteria such as *M. genomosp type 1* and *G. vaginalis* showed the higher correlation with PC1, suggesting an association of particular bacterial species with hrHPV status (Fig. 5B).

Although we observed a particular change in the CVM of samples within clusters 1 and 2, the microbiome composition was ambiguous in cluster 3, possibly due to the presence of different CST in this cluster (Fig. 5A). To further evaluate the microbial composition of our cohort, we performed a supervised average analysis comparing the CVM of hrHPV-negative (*n* = 46) and hrHPV-positive (*n* = 46) women (Additional file 21: Supplementary Figure 5). This analysis showed that hrHPV-negative women were typically colonized with *L. acidophilus*, *L. crispatus*, *L. jensenii*, *L. psittaci*, *L. ultunensis*, and *L. vaginalis*. In contrast, hrHPV-positive with high-grade lesions women possessed a more diverse microbiome with anaerobic bacteria such as *A. vaginae*, *D. micraerophilus*, *G. vaginalis*, *S. amnii*, and *S. sanguinegens*. Interestingly, *L. iners* was also present in hrHPV-positive women. Other bacteria found in hrHPV-positive women included *Prevotella* species such as *P. amnii*, *P. buccalis*, and *P. timonensis* (Additional file 21: Supplementary Figure 5). To confirm these observations, we performed a linear discriminant analysis (LDA) effect size (LEfSe) [59] modeling comparing microbiome composition and relative abundance between hrHPV-negative (*n* = 45, an outlier was excluded from this analysis) and hrHPV-positive samples (*n* = 46) (Fig. 5C). In the hrHPV-positive group, this analysis showed higher levels for *G. vaginalis*, *M. genomosp type 1*, *S. amnii*, *S. sanguinegens*, *P. anaerobius*, *D. micraerophilus*, *A. vaginae*, *P. amnii*, and *P. buccalis* (*p* < 0.05) (Fig. 5C and Additional file 22: Supplementary Figure 6A – 6I). In contrast, in the hrHPV-negative group, this analysis determined an over-representation of *L. acidophilus* (*p* < 0.05) (Fig. 5C and Additional file 22: Supplementary Figure 6 J). Thus, the alteration of the CVM due to hrHPV infection is characterized by the change from a healthy *Lactobacillus* microbiota to an anaerobic-diverse microbiota that can be explored using CiRNAseq.

### CiRNAseq profiling reveals alterations in the CVM

To further show the significance of CiRNAseq in studying CVM alterations, we examined the two clusters enriched for CST I (1) and CST IV (2) from the analysis described in Fig. 5A. We also assessed the difference in microbial richness, diversity, and relative abundance for *L. iners* in our cohort’s two main groups: hrHPV-negative women versus hrHPV-positive women with CIN2+.

**Fig. 6:**
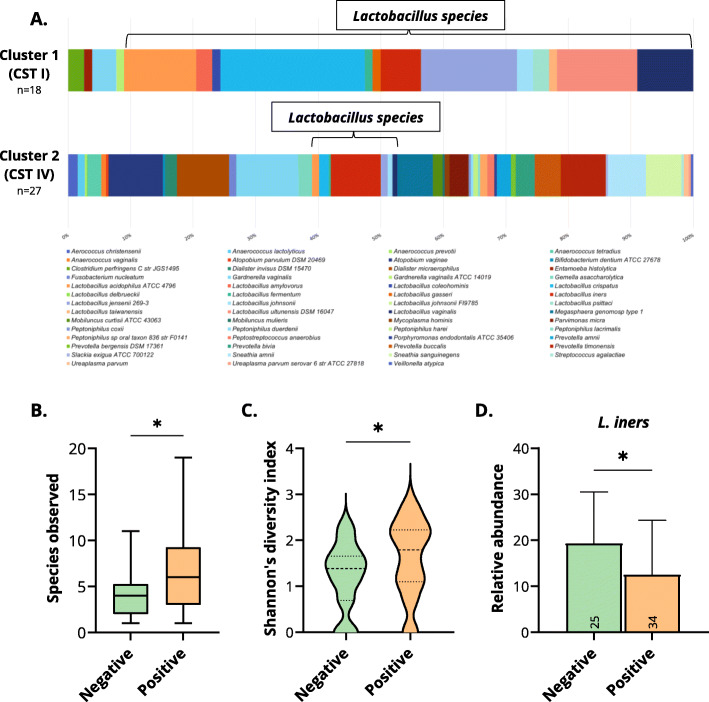
CiRNAseq profiling reveals alterations in the CVM. **A** The alteration of the microbial diversity at the species level reflects the need for high-resolution sequencing methods. Cluster 1 enriched for CST I and hrHPV-negative women has a less diverse CVM with characteristic *Lactobacillus* species. In contrast, cluster 2 enriched for CST IV and hrHPV-positive women with CIN2+ contain various microbial species in their microbiome. CST I and IV are derived from the analysis detailed in Fig. 5A. Bacterial isolates from our CVMP were considered for only three species: *G. vaginalis*, *L. johnsonii*, and *Ureaplasma parvum*. Species richness (**B**) and Shannon’s diversity index (**C**) further confirm the increase in microbial diversity in hrHPV-positive women. They also demonstrate that hrHPV infections correlate with a rich and diverse CVM. **D** Using CiRNAseq, we can quantify microbial species within the CVM. *L. iners* is less abundant in hrHPV-positive women than in hrHPV-negative women, indicating that the progression of hrHPV infections to high-grade cervical lesions is associated with a decreased relative abundance of *L. iners*. Samples were selected from our cohort of 92 samples. Negative: negative for hrHPV; Positive: positive for hrHPV; *p < 0.05

The clusters enriched for CST I and IV had 18 and 27 samples, respectively. The CVM from these two clusters seemingly varied in microbial diversity (Fig. 6A). CST I, containing mostly hrHPV-negative women, had a shallow microbial diversity characterized by *Lactobacillus* species like *L. acidophilus*, *L. crispatus*, *L. iners*, *L. jensenii*, and *L. ultunensis*. Therefore, CST I was diverse at the species level but less diverse at the genus level (Fig. 6A). In contrast, within CST IV, consisting of mainly hrHPV-positive women, such *Lactobacillus* species were depleted, and only *L. iners* continued to be present (Fig. 6A), as described in previous analyses (Fig. 5 and Additional file 21: Supplementary Figure 5). Moreover, CST IV had a highly diverse microbiome characterized by *A. vaginae*, *D. micraerophilus*, *G. vaginalis*, *L. iners*, *M. genomosp type 1*, *P. timonensis*, *S. amnii*, *S. sanguinegens*, and other bacteria as detailed in Fig. 6A. To quantify this observation, we calculated species richness and alpha-diversity, which confirmed that hrHPV-negative women had a less rich (mean of 4.2 microbes) and diverse (mean of 1.22) microbiome when compared to hrHPV-positive women, mean of 6.6 for richness and 1.60 for alpha-diversity (*p* < 0.05) (Fig. 6B, C). In conclusion, CiRNAseq let us determine that, besides a CVM change upon hrHPV infections, there is an alteration of the microbial diversity.

Given that *L. iners* colonize both hrHPV-negative and hrHPV-positive women [64, 67] but did not show a strong association to hrHPV status in our LefSe analysis, we assessed the bacterium abundance independently. To this purpose, we examined our cohort of 92 cervical samples and selected samples for which CiRNAseq identified *L. iners*. Accordingly, we included 25 hrHPV-negative samples and 34 hrHPV-positive samples for this analysis. Following the estimation of relative abundances within the samples, we calculated the mean and significance of the differences, as observed in Fig. 6D. Here, we noticed that *L. iners* had a higher relative abundance in hrHPV-negative women (mean 19.3) when compared to hrHPV-positive women (mean 11.9, *p* < 0.05), suggesting that even though it is present in the diverse microbiome of hrHPV-positive women, the abundance of this specie decreases upon infection (Fig. 6D).

## Discussion

16S rRNA gene sequencing is the most widely employed method for microbiota analysis, which have transformed community microbiological studies of the CVM [23, 28, 44, 68]. In this study, we introduce a novel targeted sequencing method with sufficient resolution and specificity to enable the profiling of cervicovaginal microbiota with similar performance to 16S rRNA-seq, but with additional advantages such as very high-throughput profiling, high taxonomic resolution, and improved sequencing sensitivity. Using CiRNAseq, we show that hrHPV-positive women with high-grade cervical intraepithelial neoplasia acquire a characteristic CST IV microbiome as observed by earlier 16S rRNA-seq studies.

CiRNAseq achieves improved sensitivity for microbiome profiling, which is a result of the underlying smMIP technique in which the same molecule is amplified multiple times over in a circular fashion. Our findings detailing the identification and quantification of genera such as *Lactobacillus*, *Gardnerella*, *Atopobium*, and *Megasphaera* with equivalent results to 16S rRNA-seq corroborate our technique’s specificity at the genus level. Nonetheless, since CiRNAseq uses two and more VRs for microbiome profiling and can target both SSU and LSU for identifying some species, its resolution increases to the species taxonomy rank, but further studies on the level of classification confidence at species resolution are warranted. Using CiRNAseq we demonstrated that in fact such genera corresponded to specific species such as *L. crispatus*, *L. iners*, *L. jensenii*, *G. vaginalis*, *A. vaginae*, or *M. genomosp type 1* which are extremely relevant for women’s cervical health and disease [4, 8, 69, 70]. Thus, our technology confirms recent studies highlighting the advantage of targeting and combining multiple VRs to improve the resolution of microbiome profiling [30, 71].

CiRNAseq showed that the CVM of women changes from a healthy-dominated *Lactobacillus* microbiome (CST I) to an anaerobic-diverse microbiome (CST IV) upon persistent hrHPV infection. Whether hrHPV infections result in a different CST or a CST IV microbiome is more permissive for hrHPV persistence is still unknown. Changes in vaginal pH have been associated with the microbial composition, particularly with depletion of *Lactobacillus* species and the enhancement of facultative anaerobic bacteria such as *G. vaginalis*, *D. micraerophilus*, *A. vaginae*, *Megasphaera* spp*.*, and *Prevotella* spp. (CST IV) [5, 72, 73]. Interestingly, Mitra et al. recently described that CST IV is highly associated with hrHPV-induced high-grade cervical intraepithelial neoplasia [15]. Since we also observe this association in our cohort of samples, it corroborates and validates the findings obtained with CiRNAseq. On the other hand, the role of individual species in the alteration of the microbiome still remains unclear. Recent studies suggest that *G. vaginalis* drives the vaginal dysbiosis in hrHPV-infected women and exhibits an immunosuppressive role in the vagina, which could explain the higher abundance of *G. vaginalis* in hrHPV-positive women described in our study [2, 64]. Therefore, identifying individual species within the CVM may elucidate the roles of particular bacteria in the microbiome and provide alternative treatment strategies to prevent disease [74]. Furthermore, understanding the CVM change at this taxonomic rank may lead to identifying microbiome profiles that could act as predictive biomarkers for women at risk of developing cervical cancer [15, 16, 18, 63, 75]. Additional studies with a larger cohort of samples are needed to clarify whether the species or CST described in the current study possess such function and explain how they would associate with the effect of hrHPV infections.

Using CiRNAseq, we also determined that hrHPV-positive women with CIN2+ have a more diverse CVM than hrHPV-negative women. In addition, our data suggest that several *Lactobacillus* species colonize hrHPV-negative women, and thus, they are more diverse at the species level than at the genus level, as previously reported [76]. Interestingly, we observed that *L. acidophilus* was highly abundant in hrHPV-negative women considered healthy, which could be attributed to the vaginal acidic conditions suitable for growth and its well-known antimicrobial activities [77, 78]. Additionally, we found higher levels of *L. iners* within the CVM of hrHPV-positive women. This finding is in line with previous research reporting that *L. iners* may not be as protective as other *Lactobacillus* species because particular *L. iners* strains have been associated with vaginal dysbiosis [3, 14]. Some studies suggest that D-lactate, produced by *L. crispatus* and not *L. iners*, enhances the trapping of HIV in the cervicovaginal mucus [11, 79]. By this mechanism, *L. crispatus*, but not *L. iners*, could also protect the basal epithelium from infection with hrHPV. Furthermore, the lower abundance of *L. iners* in smears from women with hrHPV-induced high-grade lesions could also be attributed to changes in the vaginal pH and a decline in the metabolic activities of *L. iners* [70, 73]. As far as we know, this is the first study to report a higher abundance of *L. acidophilus* in hrHPV-negative women and a lower abundance of *L. iners* in hrHPV-positive women with high-grade cervical intraepithelial neoplasia. Further studies are needed to investigate how the relative abundances of both *L. acidophilus* and *L. iners* species associate to hrHPV-induced malignancy [80].

The strengths of this study and CiRNAseq technique are the improvements to CVM profiling by targeting and combining multiple VRs and achieving higher taxonomic resolution. Potential limitations are our cohort size and the absence of testing CiRNAseq with well-defined and a high-complexity mock microbial community samples as controls [81]. Additionally, due to the use of a reference database with known microbial species, the method could have missed species that are not currently identified or suffered mutations [82, 83]. Moreover, designed probes may not hybridize to the template that carries a mismatch (e.g., diversity within species), which is why we have on average a set of six smMIPs to target and annotate one specific microbe. Worth mentioning, even though our set of 30 smMIPs can target 434 microbes, microbial species that use one smMIP for identification (e.g., viruses) were not included in our final CVMP neither annotated in this study. However, since one of the benefits is the straightforward design of smMIPs, we could add species-specific smMIPs to our pool to broaden the list of detectable species.

## Conclusions

In summary, CiRNAseq is a highly promising technology with the resolution and specificity for high-throughput sequencing, which makes it an interesting tool for uncovering the role of the CVM in health and disease. This study analyzed two outermost groups: hrHPV-negative women with no cervical lesions and hrHPV-positive women with associated high-grade cervical lesions. An obvious question is how the CVM behaves in hrHPV-positive women with no cervical lesions, low-grade cervical lesions, high-grade cervical lesions, and cancer, and such studies are on the way. Moreover, if there are “protective” or “pathogenic” species or CST associated with particular outcomes of hrHPV infections is still unresolved [84]. Future studies using CiRNAseq should allow us to evaluate the CST shift and the consequent alteration of the microbial diversity and high-resolution composition. Whether hrHPV virus or microbial species drive the CST shift is an exciting question to solve in the next studies. Nevertheless, since the microbiome depends on several factors such as hrHPV, the hrHPV genotype, the vaginal environment, and the host immune system, it is plausible that it may be more than one feature driving these microbial changes. Notably, CiRNAseq not only accomplishes quantitative microbiome profiling on the species level, but also achieves detection of viral RNAs and host gene expression products, which may allow investigations of host-microbiome interactions in a single test [35]. Overall, our work indicates that by distinguishing bacteria in high-resolution using CiRNAseq, we could further understand the association of the CVM and hrHPV infections and elucidate their potential role on cervical lesions' progression to cancer.

## Supplementary Information

**Additional file 1.** List of validated microbes with identifiers (.xlsx). List of 434 microbes selected for the in silico design and validation of smMIPs.

**Additional file 2.** List of smMIPs and sequences used for CiRNAseq profiling of the cervicovaginal microbiome (.pdf). List of designed 30 smMIPs with ligation and extension arms that target 434 relevant microbes in the cervicovaginal microbiome at the DNA and RNA levels using CiRNAseq.

**Additional file 3.** Cervicovaginal microbiome panel (.xlsx). First sheet. CVMP: list of 321 microbes profiled by CiRNAseq. Second sheet. CVMP – with isolates: list of 321 microbes including bacteria isolates. Third sheet. Common genera with 16S rRNA-seq: list of 38 genera analyzed to compare 16S rRNA-seq and CiRNAseq.

**Additional file 4.** Supplementary Table 1. Bacterial species used for in vitro experiments. MMB: Department of Medical Microbiology, Radboudumc.

**Additional file 5 **Supplementary Table 2. *E. coli* growth experiment. T: time; TP: time point of analyses; OD: optical density; RNAc: RNA concentration in ng/uL; URC: unique read counts; R1: replicate 1; R2: replicate 2; URCm: mean of URC from R1 and R2.

**Additional file 6 **Supplementary Figure 1. In silico validation of CiRNAseq specificity towards cervicovaginal microbial species. CiRNAseq targets multiple 16S rRNA gene VRs e.g. V5 – V9 for microbiome profiling. For reads assigning and species annotation, the method has a threshold of 95% of similarity between sequences and reference ROIs (**A**). Phylogenetic analyses of the 16S RNA gene for the species *Anaerococcus vaginalis*, *Anaerococcus tetradius*, *Peptostreptococcus anaerobius*, *Gardnerella vaginalis*, and *Prevotella buccalis* shows the similarity between the 16S rRNA genomes (**B**). Alignment analyses of the ROIs from the closest related species to the least related species, according to (**B**), demonstrate the specificity of CiRNAseq. For sequencing *A. vaginalis* and *A. tetradius* the technique uses the same set of five smMIPs, but two out of five ROIs exhibit <95% sequence similarity and thus do not fulfill the threshold for reads assigning (**C**). For *A. tetradius* and *P. anaerobius*, the technique uses the same four smMIPs, with their respective ROIs showing <90% sequence similarity (**D**). For *P. anaerobius* and *G. vaginalis*, the technique uses the same two smMIPs, with their ROIs having <85% sequence similarity (**E**). For *G. vaginalis* and *P. buccalis*, CiRNAseq employs the same two smMIPs, with their ROIs holding <75% sequence similarity (**F**). Marks (*) indicate smMIPs and ROIs that are dissimilar per bacterium and therefore were not included in the analyses. Unique smMIPs within the set per species increase the specificity and sensitivity of CiRNAseq for CVM profiling.

**Additional file 7 **Dataset File 1. In vitro experiment (.xlsx). First sheet. Part A: mixed bacteria and negative control. Second sheet. Part B: mixed Prevotella *spp.*, Lactobacillus *spp.*, and negative control. Values represent the percentage (%) of reactive smMIPs in each species.

**Additional file 8.** Raw data File 1. In vitro experiment A (.xlsx). Raw data from SeqNext following CiRNAseq for the in vitro experiment, part A. Values represent URC.

**Additional file 9.** In silico alignment analyses (.pdf). Results obtained with Clustal Omega v1.2.4 for Supplementary Figures 1 and 3.

**Additional file 10.** Raw data File 2. In vitro experiment B (.xlsx). Raw data from SeqNext following CiRNAseq for the in vitro experiment, part B. Values represent URC.

**Additional file 11 **Supplementary Figure 2. *E. coli* growth experiment. **A** Two replicates of the samples subjected to CiRNAseq shows the reproducibility of the technique by the number of unique read counts (URC) obtained in each replicate. **B** Mean of the replicates’ URC. **C** OD and RNA concentrations analyzed in time points five (growth), eight (autoclaved), and nine (antibiotic) were comparable with each other. However, as expected, the *E. coli* growth sample from time point eight had no URC after autoclavation, while the sample treated with cefoxitin had low URC, suggesting inhibition of bacterial metabolic activities. OD and RNA concentrations were measured before autoclavation and antibiotic treatment. T: time in hours; OD: optical density; URC: unique read counts; ng/μL: RNA concentration. *, *p* <0.05; **, *p* <0.01; ***, *p* <0.001; ****, *p* <0.0001; NS, not significant.

**Additional file 12 **Supplementary Figure 3. CiRNAseq specificity towards *Bifidobacterium* and *Gardnerella* species. 16S rRNA-seq targets two variable regions (VRs) of the 16S rRNA gene using a forward and a reverse primer (e.g., V3 and V4). Alternatively, CiRNAseq targets five VRs of the 16S subunit using five singular smMIPs to differentiate species of *B.longum* and *G. vaginalis*. There is a high percentage of similarity (>90%) when comparing the V3-V4 regions of *B. longum* and *G. vaginalis*, which, if it is not appropriately amplified, could result in misidentification. In contrast, the CiRNAseq ROIs for both species have different levels of identity. Two out of five ROIs also possess a high percentage of similarity (>90%), with both amplifying the V7 and V8 VRs, and needed to identify these microbes at the family level. The rest remaining three out of five ROIs share less than 45% of similarity, which endorses the resolution and specificity of CiRNAseq in detecting both species. The color red represents similarity in sequences, while the color black represents no similarity.

**Additional file 13.** Raw data File 3. 16S rRNA-seq (.txt). Raw data from QIIME following microbiome profiling of ten cervical smears using 16S rRNA-seq. Corresponding IDs (manuscript ID = raw data ID): SN1A = KA1, SN2A = S2, SN3A = S5, SN4A = KA6, SN5A = S7, SN6A = S8, SN7A = S9.

**Additional file 14.** Raw data File 4. CiRNAseq (.xlsx). Raw data from SeqNext following microbiome profiling of eight cervical smears using CiRNAseq. The first seven sheets represent the seven samples included in the final analyses. The last sheet shows the sample with <1000 URC and excluded from the calculations. Values represent URC.

**Additional file 15.** Dataset File 2. 16S rRNA-seq vs CiRNAseq (.csv). Relative abundances for the 38 genera analyzed in seven cervical smears profiled with both 16S rRNA-seq and CiRNAseq.

**Additional file 16 **Supplementary Figure 4. Pearson’s correlation for *Lactobacillus*, *Gardnerella, Atopobium* and *Megasphaera*. Pearson’s positive correlation obtained from comparing the detection of *Lactobacillus* (**A**) (*r* = 0.9605, *p* = 0.0006), *Gardnerella* (**B**) (*r* = 0.9384, *p* = 0.0018), *Atopobium* (**C**) (*r* = 0.9255, *p* = 0.0028), and *Megasphaera* (*r* = 0.8344, *p* = 0.0196) using both CiRNAseq and 16S rRNA-seq corroborates the specificity and sensitivity of CiRNAseq.

**Additional file 17.** Dataset File 3. CiRNAseq resolution (.csv). List of all species detected with CiRNAseq in the same seven cervical smears that were initially compared to 16S rRNA-seq. Species annotation is indicated by 1. No annotation is indicated by 0.

**Additional file 18.** Raw data File 5. hrHPV cohort (.xlsx). Raw data from SeqNext following microbiome profiling of 92 cervical smears using CiRNAseq. Values represent URC.

**Additional file 19.** Dataset File 4. hrHPV cohort (.csv). Cohort of 46 hrHPV negative samples and 46 hrHPV positive samples (CIN2+), for which the microbiomes were profiled using CiRNAseq. Species annotation was performed with a custom R script. The CVMP includes bacterial isolates. Values represent URC.

**Additional file 20.** Loading scores from PCA analysis (.csv). PCA loading scores showing that anaerobic bacteria have the higher correlation with PC1.

**Additional file 21 **Supplementary Figure 5. Average analysis of hrHPV cohort. Average analysis of hrHPV negative versus hrHPV positive with CIN2+ demonstrates a strong association of absence and presence of particular microbes. *L. acidophilus*, *L. jensenii* and *L. crispatus* were highly present in hrHPV negative women, but the microbiome seemingly changes to a diverse-anaerobic microbiota in hrHPV positive women with CIN2+.

**Additional file 22 **Supplementary Figure 6. LefSe analysis: relative abundances association with hrHPV status. Relative abundance counts of *G. vaginalis* (**A**), *M. genomosp type 1* (**B**), *S. amnii* (**C**), *S. sanguinegens* (**D**), *P. anaerobius* (**E**), *D. micraerophilus* (**F**), *A. vaginae* (**G**), *P. amnii* (**H**), and *P. buccalis* (**I**) were found significantly over-represented in hrHPV positive women whereas *Lactobacillus acidophilus* (**J**) was enriched in hrHPV negative women.

## Data Availability

All data generated or analyzed during this study are included in this published article and its supplementary information files or are available from public repositories. The sequence read data generated in this study are available at EMBL in the European Nucleotide Archive, project PRJEB45937 [85].

## Abbreviations

16S rRNA-seq: 16S rRNA gene sequencing
BED: Browser Extensible Data
BWA: Burrows-Wheeler Aligner
CCSP: Cervical cancer screening program
CIN: Cervical intraepithelial neoplasia
CiRNAseq: Circular probes-based RNA sequencing
CST: Community state types
CVM: Cervicovaginal microbiome
CVMP: Cervicovaginal microbiome panel
CVMPs: Cervicovaginal microbiome panel shortened
gDNA: Genomic DNA
HVMP: Human Vaginal Microbiome Project
hrHPV: High-risk human papillomavirus
LSU: Large ribosomal subunit
NCBI: National Center for Biotechnology Information
OD: Optical density
ROIs: Region of interests
rRNA: Ribosomal RNA
smMIPs: Single-molecule molecular inversion probes
SSU: Small ribosomal subunit
UMI: Unique molecular identifier
URC: Unique read counts
VRs: Hypervariable regions
