# Supplementary material for: Novel high-resolution targeted sequencing of the cervicovaginal microbiome
Source: BMC Biol. 2021 Dec 16;19:267. doi: 10.1186/s12915-021-01204-z (PMC8680041; doi:10.1186/s12915-021-01204-z)
Supplement: Supplementary file 2 — Additional file 2. List of smMIPs and sequences used for CiRNAseq profiling of the cervicovaginal microbiome (.pdf). List of designed 30 smMIPs with ligation and extension arms that target 434 relevant microbes in the cervicovaginal microbiome at the DNA and RNA levels using CiRNAseq. [file 12915_2021_1204_MOESM2_ESM.pdf]

List of smMIPs and sequences used for CiRNAseq profiling of the cervicovaginal microbiome

ID	Working ID	Extension probe	Ligation probe
smMIP1	Anaerococcus_obesiensis+Anaerococcus_obesiensis_product_23S_0022	CAGATGGAAAAGATGGTCT	AGTCACTATCCAGACCAAAGCGCCC
smMIP2	Aspergillus_tubingensis+KT326815_1_product_18S_0002	AGAAATTGACGGAAGGGCA	GGATGGTGGTGCATGGCCGTTCTTAG
smMIP3	Bacteroides_dorei_DSM_17855+product_23S_0009	TTCTGCGTGAATCTGCCGGG	TGCGCCTACAAGCGGTCGGAGCT
smMIP4	Bacteroides_sp_3_1_23_NZ_GG774959_1_rna10_0006	AATGCGTTCCCGGGCCTTGTA	CGTAACAAGGTAGCCGTACCGGAA
smMIP5	Bifidobacterium_angulatum_JCM_7096_GCF_0010251551_ASM102515v1_NZ_AP012322_1_rna0_0019	TGGCTAGTTGTAGAGAGTAGTAAAA	GTAGTCATATGCTTGTCTC
smMIP6	Candida_orthopsilosis_Candida_orthopsilosis_product_18S_0028	CTGGGGGATAACAGTTAG	ACGGCCCACCAAAGCGACGATCAGTAG
smMIP7	Clostridium_bolteae_ATCC_BAA_613_GCF_0001543651_ASM15436v1_NZ_DS480694_1_rna30_0007	ACCGCCCGTCGCTACTACCG	CAAGGTTTCCGTAGGTGAACC
smMIP8	Coprinus_comatus_Coprinus_comatus_product_18S_0002	AATTGACGGGGCCCCGCACAAGC	GGTGGTGCATGGCTGTCGTC
smMIP9	Cutibacterium_acnes_J139_NZ_ADFS01000001_1_rna32_0028	CCCAGGCAACTGTTTATCAAAA	CGGCGGCCGTAACTATAACGGT
smMIP10	Eubacterium_siraeum_DSM_15702_GCF_0003820851_ASM38208v1+NZ_KB907544_1__0016	TGTACAGGATAGGTGGGAGA	GTGGGCAGTTTGACTGGGGCGGT
smMIP11	Eubacterium_siraeum_DSM_15702_GCF_0003820851_ASM38208v1+NZ_KB907544_1__0020	TAGCAAACAGGATTAGA	GCAAGGTTGAAACTCAAAGGAATTGACG
smMIP12	Faecalibacterium_prausnitzii_NZ_DS483500_1_rna34_0106	GTTGTCACGCCAGTGG	GATAGGTTGGGGGTGTACGCGCAGTAAT
smMIP13	Fusobacterium_equinum+product_23S_0034	TTACGCGTTACTCACCCGTC	CACTGTTTTGATTTTTTACACCTTG
smMIP14	Fusobacterium_gonidiaformans+NZ_GG657972_1_rna44_0002	ATGGGGCAAAAGACGT	GGTTTTCTGCGTTCAGCCTGAGAAGGGGG
smMIP15	Hepacivirus_C_NC_004102_1_id2_0121	CCACAAGAACACATCATACAA	GTGGTTATCCTGCGTTGATGCCT
smMIP16	Hepatitis_B_virus_NC_003977_2_cds6_0017	CTCTGTGTTAGTTTAAAGTGCA	GTAAGGTGCAGAAAGAATATGCA
smMIP17	Human_immunodeficiency_virus_NC_001802_1_id7_0072	GCGAAAGCGTGGGTAG	GGGGAGTACGGCCGCAAGGTTGAAACT
smMIP18	Mageeibacillus_indolicus_NC_013895_2_rna7_0011	TGATTTCTCGTAAGGTGCCG	AATGAAAACGTCCTTGGCAAATGC
smMIP19	Nakaseomyces_delphensis+Nakaseomyces_delphensis_product_18S_0023	TGCTGATTTGACGTCATCCCCACCT	GCACGAGCTGACGACAACCA
smMIP20	Prevotella_copri_NZ_GG703855_1_rna43_0029	GGAAGGTGCGGCTGGA	GGGGGATTAGCTCAGTTGGCTAGA
smMIP21	Prevotellamassilia_timonensis_NZ_LT629844_1_rna35_0020	TCCAAAGGAACTCCTACCTTACGCC	CCCTGTCACAGAACTGCCGC
smMIP22	Primate_T_lymphotropic_virus_1_NC_001436_1_id2_0064	CCTTCAACAGGACATCA	GACTCTAAATATTTAATCAAATACCT
smMIP23	Primate_T_lymphotropic_virus_2_NC_001488_1_id8_0071	AAACCAACCGGGATTGCCTT	TGGAACAGGACGTCATAGAG
smMIP24	Saccharomyces_pastorianus_Saccharomyces_pastorianus_product_26S_0001	CTTCATGTAGTCGAGTTGCAGA	GCATGATGATTTGACGTCATCCC
smMIP25	Shuttleworthia_satelles_DSM_14600_NZ_GG665866_1_rna19_0005	AAGTCGTAACAAGGTAGCC	GGAGAGCGCCTGCTTTGCACGCAGGA
smMIP26	Streptococcus_gordonii_str_Challis_substr_CH1+NC_009785_1_rna27_0043	TCCAATAGTTATCCCC	CAGCGTTCGTCCTGAGCCAGGATCAAACT
smMIP27	Streptococcus_macedonicus_NC_016749_1_rna66_0143	AGCTAATACCGCATAA	GATACATAGCCGACCTGAGAGGGTGATCG
smMIP28	Streptococcus_macedonicus+NC_016749_1_rna0_0019	TTGAAAACTGAACAAGAC	TTTGATCCTGGCTCAGGACGAACGC
smMIP29	Streptococcus_oralis_subsp_dentisani+NZ_CAUJ01000005_1_rna23_0027	GGACGAAAGTCTGACGGAG	GCCACGGCTAACTACGTGCCAGCAG
smMIP30	Veillonella_seminalis_NZ_JH992937_1_rna41_0017	TCGACTTGCATGTGTTAAGC	TGACCTTATTCAAATACCTA
