# Supplementary material for: Novel high-resolution targeted sequencing of the cervicovaginal microbiome
Source: BMC Biol. 2021 Dec 16;19:267. doi: 10.1186/s12915-021-01204-z (PMC8680041; doi:10.1186/s12915-021-01204-z)
Supplement: Supplementary file 4 — Additional file 4. Supplementary Table 1. Bacterial species used for in vitro experiments. MMB: Department of Medical Microbiology, Radboudumc. [file 12915_2021_1204_MOESM4_ESM.pdf]

**Bacterial species**	**Source**	**Authentication reference**
Anaerococcus tetradius	MMB	Sanger sequencing
Anaerococcus vaginalis	MMB	Sanger sequencing
Escherichia coli	Commercial	ATCC 25922
Gardnerella vaginalis	MMB	Sanger sequencing
Peptostreptococcus anaerobius	MMB	Sanger sequencing
Prevotella buccalis	MMB	Sanger sequencing
Prevotella copri	MMB	Sanger sequencing
Prevotella denticola	MMB	Sanger sequencing
Prevotella disiens	MMB	Sanger sequencing
Lactobacillus delbruecki	MMB	Sanger sequencing
Lactobacillus fermentum	MMB	Sanger sequencing
Lactobacillus jensenii	MMB	Sanger sequencing
