# Supplementary material for: Novel high-resolution targeted sequencing of the cervicovaginal microbiome
Source: BMC Biol. 2021 Dec 16;19:267. doi: 10.1186/s12915-021-01204-z (PMC8680041; doi:10.1186/s12915-021-01204-z)
Supplement: Supplementary file 5 — Additional file 5 Supplementary Table 2. E. coli growth experiment. T: time; TP: time point of analyses; OD: optical density; RNAc: RNA concentration in ng/uL; URC: unique read counts; R1: replicate 1; R2: replicate 2; URCm: mean of URC from R1 and R2. [file 12915_2021_1204_MOESM5_ESM.pdf]

T	TP	OD	RNAc	URC R1	URC R2	URCm	Comment
0	1	0.057	3.26	345	594	469.5	Lag phase
1	-	0.057	-	-	-	-	-
2	2	0.061	2.82	600	488	544	Start growth phase
3	-	0.062	-	-	-	-	-
4	3	0.078	6.12	1257	1303	1280	Growing phase
5	4	0.145	11.45	1713	1369	1541	Begin of exponential phase
6	5	0.206	15.7	1959	1895	1927	Exponential + growing phase
7	-	0.280	-	-	-	-	-
22.5	6	0.349	18.15	1213	943	1078	Stationary phase
23.5	7	0.315	15.95	1160	1268	1214	Stationary phase
24.5	-	0.292	-	-	-	-	-
25.5	-	0.345	-	-	-	-	-
26.5	8	0.317	8.86	0	0	0	Autoclaved
27.5	-	0.290	-	-	-	-	-
28.5	-	0.308	-	-	-	-	-
29.5	-	0.335	-	-	-	-	-
46.5	-	0.305	-	-	-	-	-
47.5	-	0.296	-	-	-	-	-
48.5	9	0.301	11.65	723	1648	1185.5	Antibiotic
