# Supplementary figures and images for: Novel high-resolution targeted sequencing of the cervicovaginal microbiome
Source: BMC Biol. 2021 Dec 16;19:267. doi: 10.1186/s12915-021-01204-z (PMC8680041; doi:10.1186/s12915-021-01204-z)

**A.**

16S rRNA gene

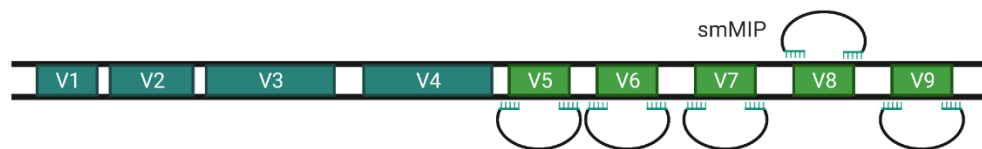

**B.**

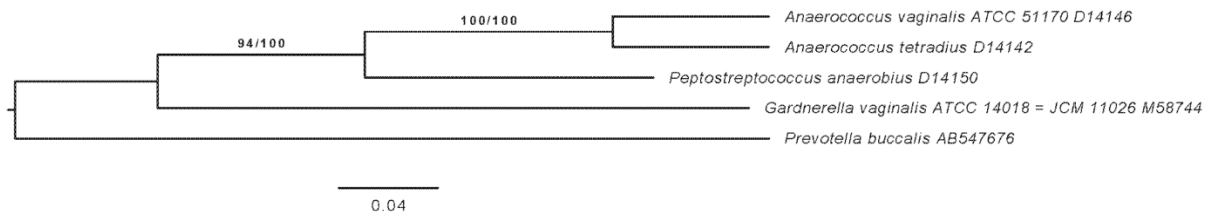

**C.**

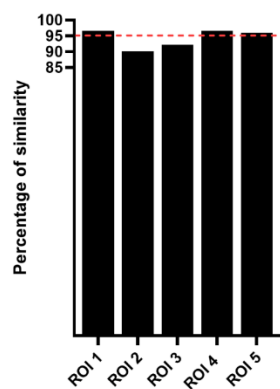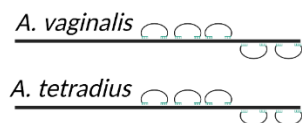

**D.**

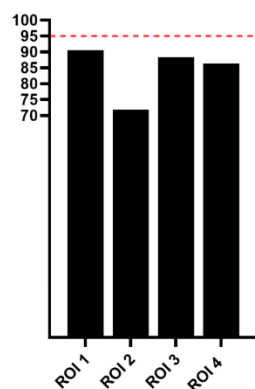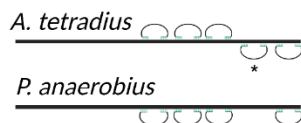

**E.**

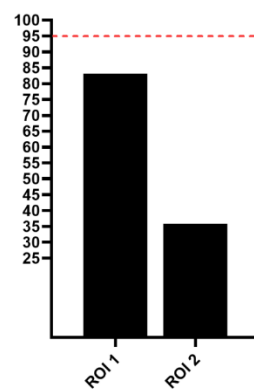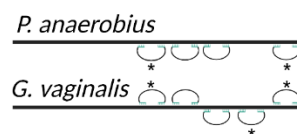

**F.**

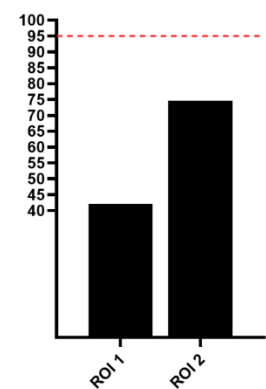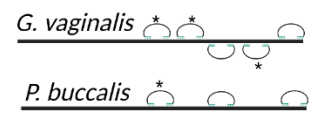

Supplement: Supplementary file 6 — Additional file 6 Supplementary Figure 1. In silico validation of CiRNAseq specificity towards cervicovaginal microbial species. CiRNAseq targets multiple 16S rRNA gene VRs e.g. V5 – V9 for microbiome profiling. For reads assigning and species annotation, the method has a threshold of 95% of similarity between sequences and reference ROIs (A). Phylogenetic analyses of the 16S RNA gene for the species Anaerococcus vaginalis, Anaerococcus tetradius, Peptostreptococcus anaerobius, Gardnerella vaginalis, and Prevotella buccalis shows the similarity between the 16S rRNA genomes (B). Alignment analyses of the ROIs from the closest related species to the least related species, according to (B), demonstrate the specificity of CiRNAseq. For sequencing A. vaginalis and A. tetradius the technique uses the same set of five smMIPs, but two out of five ROIs exhibit <95% sequence similarity and thus do not fulfill the threshold for reads assigning (C). For A. tetradius and P. anaerobius, the technique uses the same four smMIPs, with their respective ROIs showing <90% sequence similarity (D). For P. anaerobius and G. vaginalis, the technique uses the same two smMIPs, with their ROIs having <85% sequence similarity (E). For G. vaginalis and P. buccalis, CiRNAseq employs the same two smMIPs, with their ROIs holding <75% sequence similarity (F). Marks (*) indicate smMIPs and ROIs that are dissimilar per bacterium and therefore were not included in the analyses. Unique smMIPs within the set per species increase the specificity and sensitivity of CiRNAseq for CVM profiling. [file 12915_2021_1204_MOESM6_ESM.pdf]

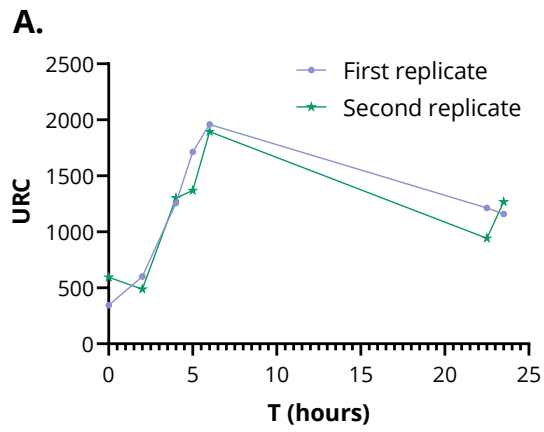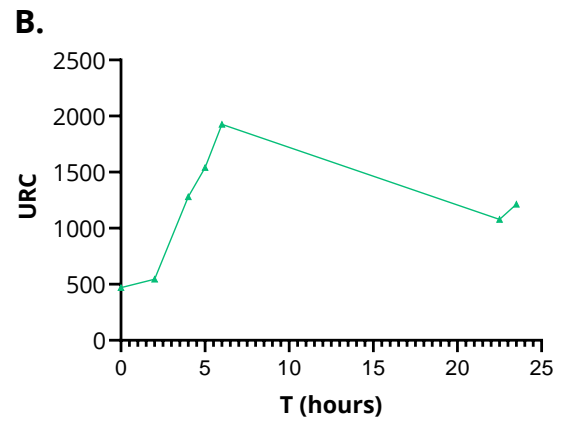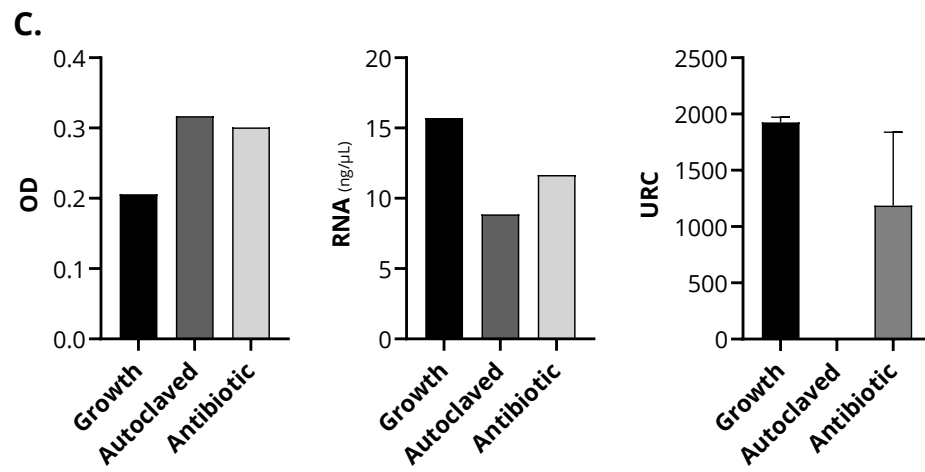

Supplement: Supplementary file 11 — Additional file 11 Supplementary Figure 2. E. coli growth experiment. A Two replicates of the samples subjected to CiRNAseq shows the reproducibility of the technique by the number of unique read counts (URC) obtained in each replicate. B Mean of the replicates’ URC. C OD and RNA concentrations analyzed in time points five (growth), eight (autoclaved), and nine (antibiotic) were comparable with each other. However, as expected, the E. coli growth sample from time point eight had no URC after autoclavation, while the sample treated with cefoxitin had low URC, suggesting inhibition of bacterial metabolic activities. OD and RNA concentrations were measured before autoclavation and antibiotic treatment. T: time in hours; OD: optical density; URC: unique read counts; ng/μL: RNA concentration. *, p <0.05; **, p <0.01; ***, p <0.001; ****, p <0.0001; NS, not significant. [file 12915_2021_1204_MOESM11_ESM.pdf]

16S rRNA  
gene sequencing

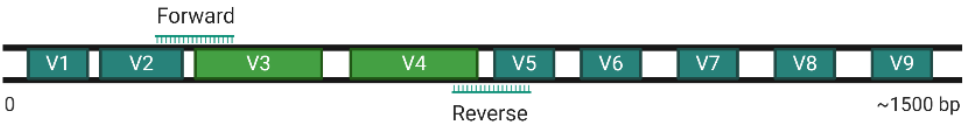

CiRNAseq

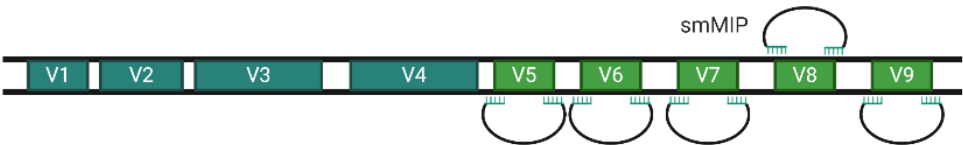

16S *B. longum*  
16S *G. vaginalis*

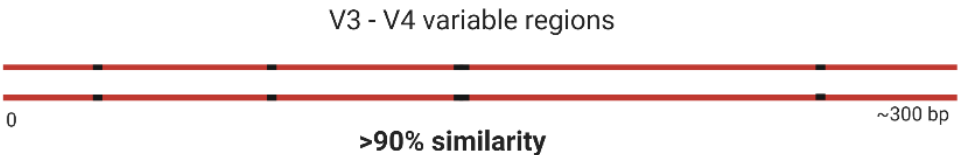

ROIs for *B. longum*  
ROIs for *G. vaginalis*

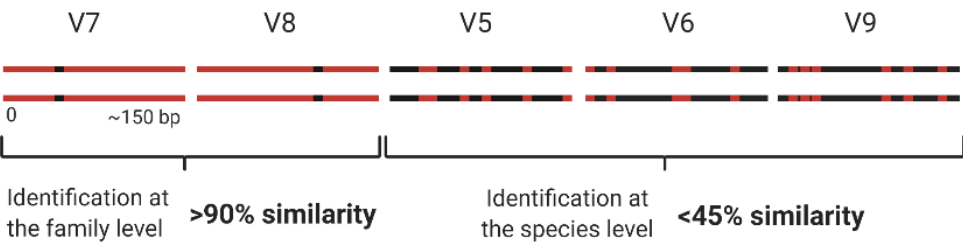

Supplement: Supplementary file 12 — Additional file 12 Supplementary Figure 3. CiRNAseq specificity towards Bifidobacterium and Gardnerella species. 16S rRNA-seq targets two variable regions (VRs) of the 16S rRNA gene using a forward and a reverse primer (e.g., V3 and V4). Alternatively, CiRNAseq targets five VRs of the 16S subunit using five singular smMIPs to differentiate species of B.longum and G. vaginalis. There is a high percentage of similarity (>90%) when comparing the V3-V4 regions of B. longum and G. vaginalis, which, if it is not appropriately amplified, could result in misidentification. In contrast, the CiRNAseq ROIs for both species have different levels of identity. Two out of five ROIs also possess a high percentage of similarity (>90%), with both amplifying the V7 and V8 VRs, and needed to identify these microbes at the family level. The rest remaining three out of five ROIs share less than 45% of similarity, which endorses the resolution and specificity of CiRNAseq in detecting both species. The color red represents similarity in sequences, while the color black represents no similarity. [file 12915_2021_1204_MOESM12_ESM.pdf]

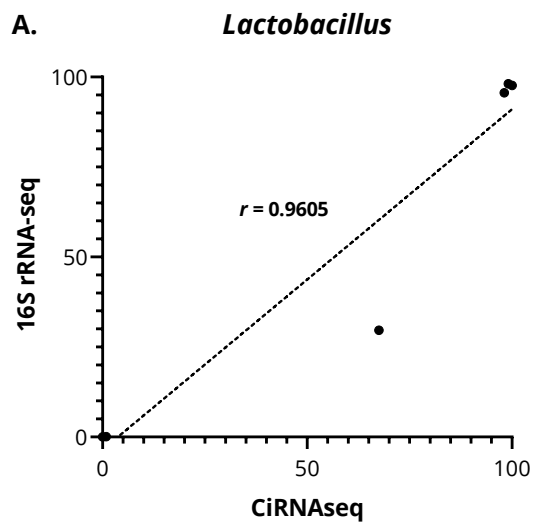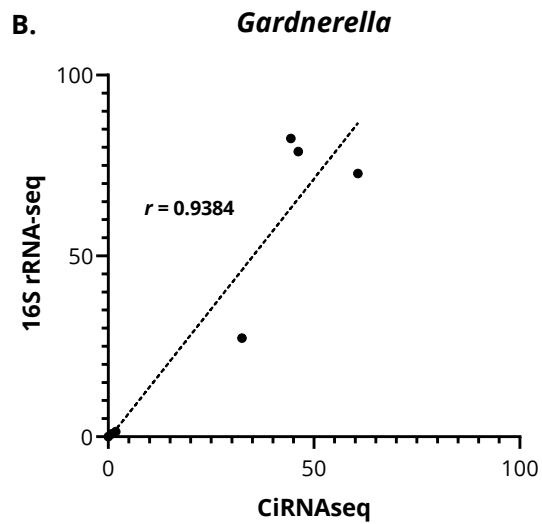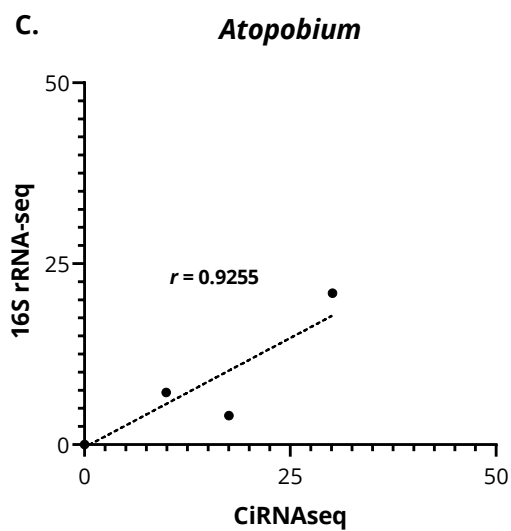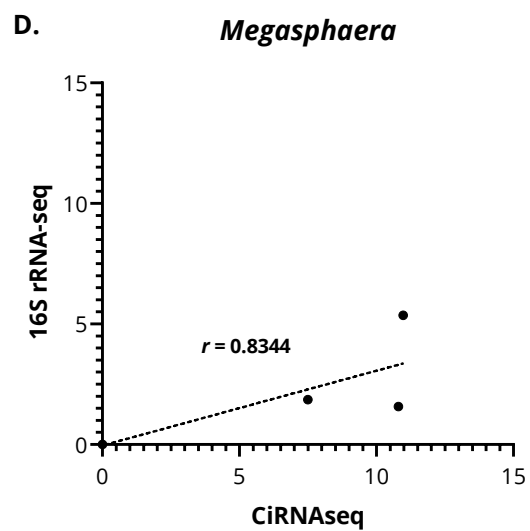

Supplement: Supplementary file 16 — Additional file 16 Supplementary Figure 4. Pearson’s correlation for Lactobacillus, Gardnerella, Atopobium and Megasphaera. Pearson’s positive correlation obtained from comparing the detection of Lactobacillus (A) (r = 0.9605, p = 0.0006), Gardnerella (B) (r = 0.9384, p = 0.0018), Atopobium (C) (r = 0.9255, p = 0.0028), and Megasphaera (r = 0.8344, p = 0.0196) using both CiRNAseq and 16S rRNA-seq corroborates the specificity and sensitivity of CiRNAseq. [file 12915_2021_1204_MOESM16_ESM.pdf]

**A.**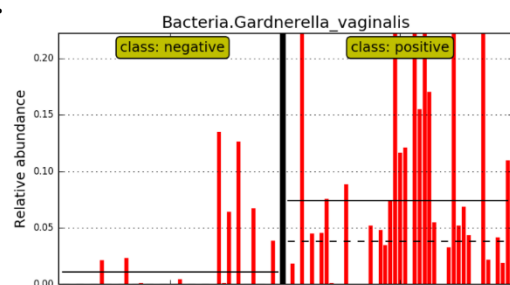**B.**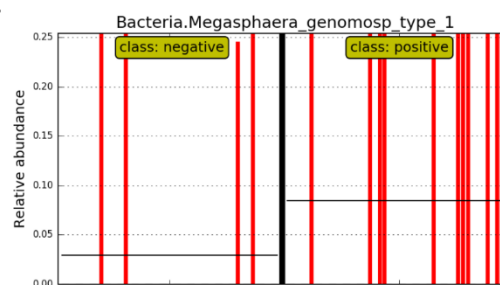**C.**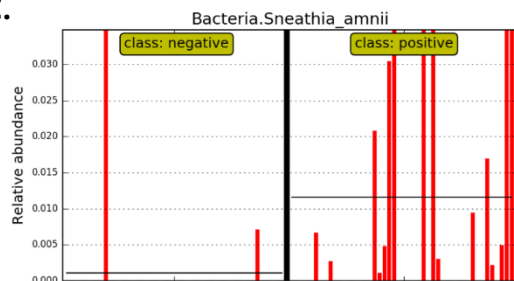**D.**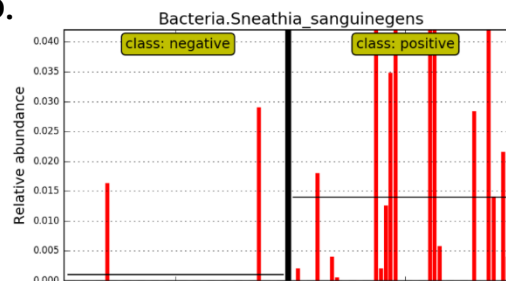**E.**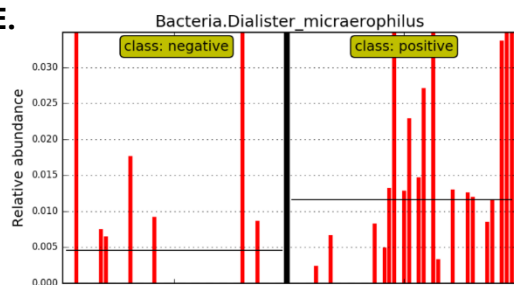**F.**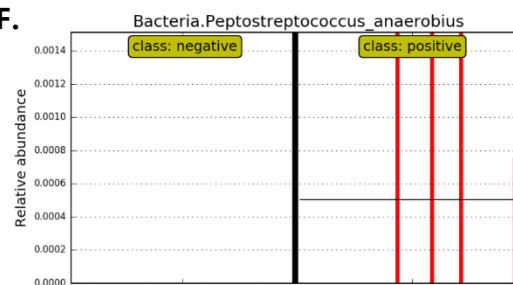**G.**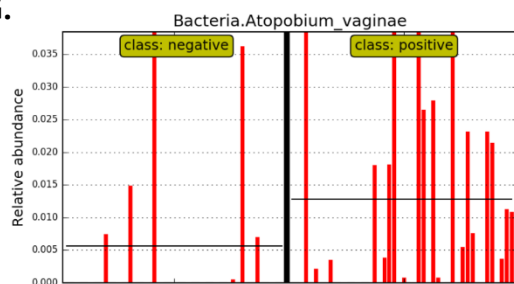**H.**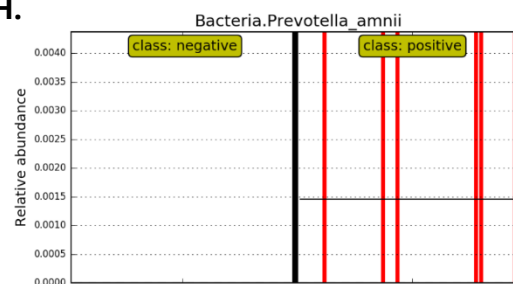**I.**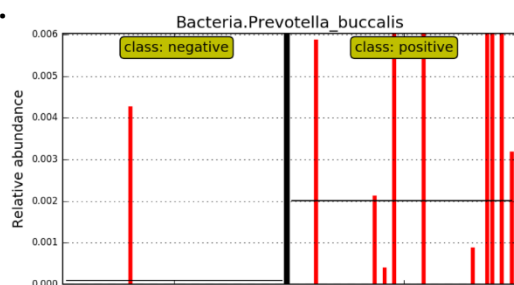**J.**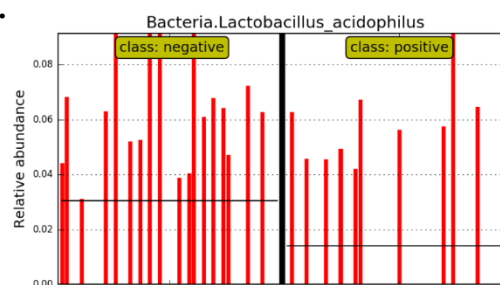

Supplement: Supplementary file 22 — Additional file 22 Supplementary Figure 6. LefSe analysis: relative abundances association with hrHPV status. Relative abundance counts of G. vaginalis (A), M. genomosp type 1 (B), S. amnii (C), S. sanguinegens (D), P. anaerobius (E), D. micraerophilus (F), A. vaginae (G), P. amnii (H), and P. buccalis (I) were found significantly over-represented in hrHPV positive women whereas Lactobacillus acidophilus (J) was enriched in hrHPV negative women. [file 12915_2021_1204_MOESM22_ESM.pdf]
