# Supplementary material for: Novel high-resolution targeted sequencing of the cervicovaginal microbiome
Source: BMC Biol. 2021 Dec 16;19:267. doi: 10.1186/s12915-021-01204-z (PMC8680041; doi:10.1186/s12915-021-01204-z)
Supplement: Supplementary file 21 — Additional file 21 Supplementary Figure 5. Average analysis of hrHPV cohort. Average analysis of hrHPV negative versus hrHPV positive with CIN2+ demonstrates a strong association of absence and presence of particular microbes. L. acidophilus, L. jensenii and L. crispatus were highly present in hrHPV negative women, but the microbiome seemingly changes to a diverse-anaerobic microbiota in hrHPV positive women with CIN2+. [file 12915_2021_1204_MOESM21_ESM.pdf]

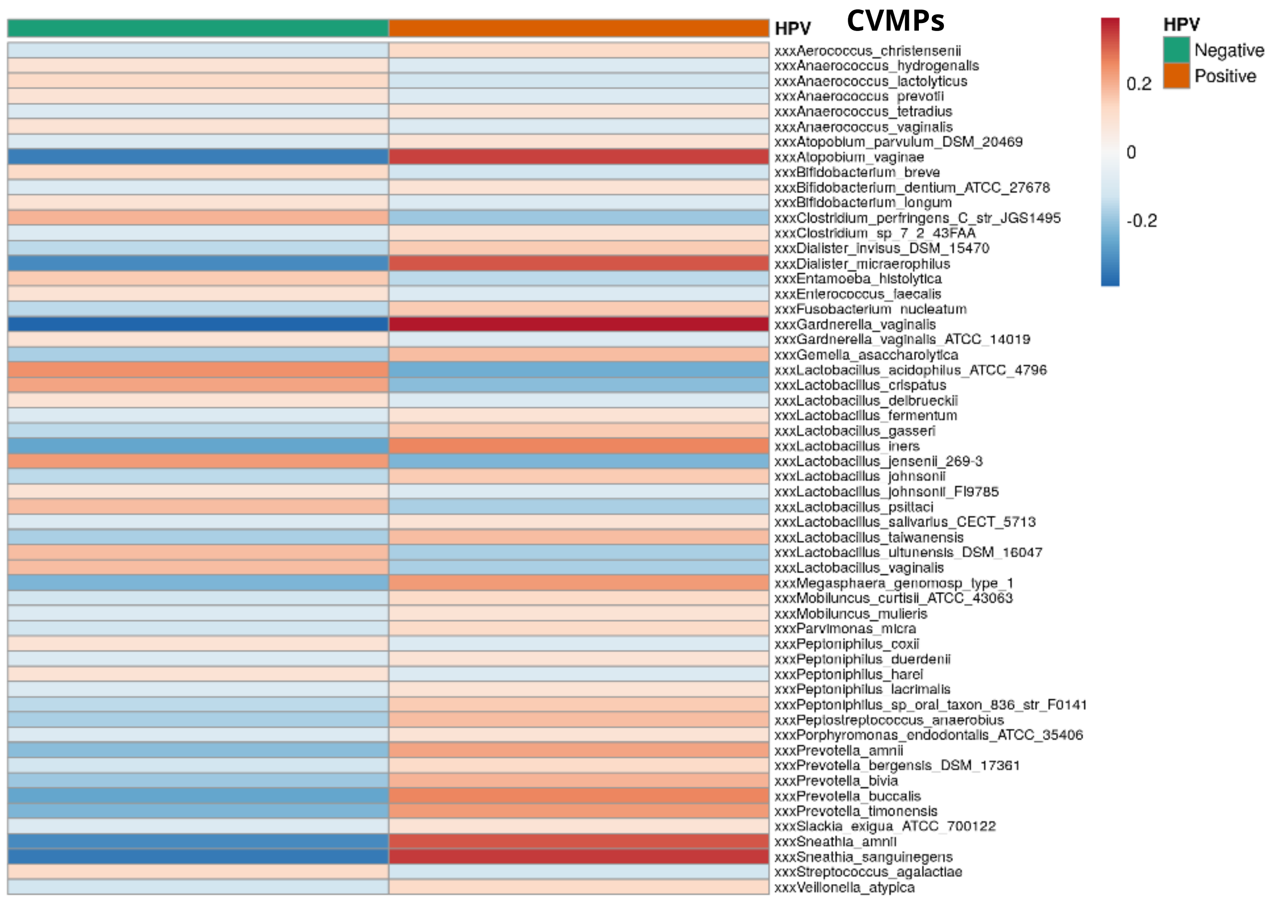

CVMPs: cervicovaginal microbiome panel shortened.
