# Supplementary material for: Novel high-resolution targeted sequencing of the cervicovaginal microbiome
Source: BMC Biol. 2021 Dec 16;19:267. doi: 10.1186/s12915-021-01204-z (PMC8680041; doi:10.1186/s12915-021-01204-z)
Supplement: Supplementary file 9 — Additional file 9. In silico alignment analyses (.pdf). Results obtained with Clustal Omega v1.2.4 for Supplementary Figures 1 and 3. [file 12915_2021_1204_MOESM9_ESM.pdf]

In silico analyses I

1. Anaerococcus vaginalis vs. Anaerococcus tetradius

ROI 1

```
CLUSTAL O(1.2.4) multiple sequence alignment


Anaerococcus_tetradius_smMIPPcopri      TGATGATTTGACGTCATCCCCACCTTCCTCCGGTTTATCACCGGCAGTATATCTAGAGTC    60
Anaerococcus_vaginalis_smMIPPcopri      TGATGATTTGACGTCGTCCCCACCTTCCTCCGGTTTATCACCGGCAGTATCGCTAGAGTC    60
                                        *************** **********************************  ********

Anaerococcus_tetradius_smMIPPcopri      CCCAACTTAATGATGGTAACTAAATATAGGGGTTGCGCTCGTTATGGGACTTAACCCAAC    120
Anaerococcus_vaginalis_smMIPPcopri      CCCAACTTAATGATGGTAACTAACGATAGGGGTTGCGCTCGTTATGGGACTTAACCCAAC    120
                                        ***********************  ***********************************

Anaerococcus_tetradius_smMIPPcopri      ATCTCACGACACGAGCTGACGACAACCA    148
Anaerococcus_vaginalis_smMIPPcopri      ATCTCACGACACGAGCTGACGACAACCA    148
                                        ****************************
```

5 SNPs on 148 nt = (148-5: 143)/148 * 100 = **96.62% similarity**

ROI 2

```
CLUSTAL O(1.2.4) multiple sequence alignment


Anaerococcus_tetradius_smMIPSsatelles      CTTCATGCACTCGAGTTGCAGAGTGCAATCCGAACTGGGACAGGCTTTTTGAGTTTTGCT 60
Anaerococcus_vaginalis_smMIPSsatelles      CTTCATGTACTCGAGTTGCAGAGTACAATCCGAACTGGGACAGACTTTGTGAGTTTCGCT 60
                                           ******* **************** ****************** **** ******* ***

Anaerococcus_tetradius_smMIPSsatelles      TAACTTCGCAGTCTCGCTTCCCTGTGTACCTGCCATTGTAGCACGTGTGTAGCCCAAGTC 120
Anaerococcus_vaginalis_smMIPSsatelles      TCAGATCGCTCTTTCGCTGCCCTCTGTATCTGCCATTGTAGCACGTGTGTAGCCCAAGTC 120
                                           * *  ****  * ***** **** **** *******************************

Anaerococcus_tetradius_smMIPSsatelles      ATAAAGGGCATGATGATTTGACGTCATCCC      150
Anaerococcus_vaginalis_smMIPSsatelles      ATAAAGGGCATGATGATTTGACGTCGTCCC      150
                                           ************************* ****
```

15 SNPs on 150 nt = (150-15: 135)/150 * 100 = **90.00% similarity**

ROI 3

```
CLUSTAL O(1.2.4) multiple sequence alignment


Anaerococcus_tetradius_smMIPCacnes      GACGACAACCATGCACCACCTGTATTACAGTCTTCCGAAGAAGTAAGGAACTATCTCTAA    60
Anaerococcus_vaginalis_smMIPCacnes      GACGACAACCATGCACCACCTGTAATACAGTAGACCGAAGTCATAGAAAATTATCTCTAA    60
                                        ************************ ******   ******   **   ** *********

Anaerococcus_tetradius_smMIPCacnes      TTCCCTCCGTAATATGTCAAGACTTGGTAAGGTTCTTCGCGTTGCGTCGAATTAAACCAC    120
Anaerococcus_vaginalis_smMIPCacnes      TTCCCTCCGTATTATGTCAAGACTTGGTAAGGTTCTTCGCGTTGCGTCGAATTAAACCAC    120
                                        *********** ************************************************

Anaerococcus_tetradius_smMIPCacnes      ATGCTCCGCTGCTTGTGCGGGTCCCCGTCAATT      153
Anaerococcus_vaginalis_smMIPCacnes      ATGCTCCGCTGCTTGTGCGGGTCCCCGTCAATT      153
                                        *********************************
```

12 SNPs on 153 nt = (153-12: 141)/153 * 100 = **92.16% similarity**

ROI 4

```
CLUSTAL O(1.2.4) multiple sequence alignment


Anaerococcus_tetradius_smMIPBangulatum      TTCCGATACGGCTACCTTGTTACGACTTCACCCCAGTTACTGACCCTACCTTCGACTGCT      60
Anaerococcus_vaginalis_smMIPBangulatum      TTCCGATACGGCTACCTTGTTACGACTTCACCCCAGTTACTGACCCTACCTTCGACTGCT      60
                                            ************************************************************

Anaerococcus_tetradius_smMIPBangulatum      GCTTCCTAA-TGGTTAGCTCACAGGCTTCGGGTATTGCCAACTTCCATGGTGTGACGGGC      119
Anaerococcus_vaginalis_smMIPBangulatum      GCGTCCTAAAAGGTTCGCTCACAGGCTTCGGGTATTGCCAACTCCCATGGTGTGACGGGC      120
                                            ** ******  **** *************************** ****************

Anaerococcus_tetradius_smMIPBangulatum      GGTGTGTACAAGACCCGGGAACGCATT 146
Anaerococcus_vaginalis_smMIPBangulatum      GGTGTGTACAAGACCCGGGAACGCATT 147
                                            ***************************
```

5 SNPs on 146 nt = (146-5: 141)/146 * 100 = **96.58% similarity**

ROI 5

```
CLUSTAL O(1.2.4) multiple sequence alignment


Anaerococcus_tetradius_smMIPFpraustnizii      CGTCAATTCCTTTGAGTTTCACACTTGCGTGCGTACTCCCCAGGCGGAGTGTTTATTGCG      60
Anaerococcus_vaginalis_smMIPFpraustnizii      CGTCAATTCCTTTGAGTTTCACACTTGCGTGCGTACTCCCCAGGCGGAGTGTTTAATGCG      60
                                              ******************************************************* ****

Anaerococcus_tetradius_smMIPFpraustnizii      TTAGCTGCGGCACCCAGATTATTCCAGACACCTAACACTCATCGTTTACGGCGTGGACTA      120
Anaerococcus_vaginalis_smMIPFpraustnizii      TTAGCTGCGGCTCCCAGATTATTCCAAGAACCTAACACTCATCGTTTACAGCGTGGACTA      120
                                              *********** **************   ******************** **********

Anaerococcus_tetradius_smMIPFpraustnizii      CCAGGGTATCTAATCCTGTTTGCTA 145
Anaerococcus_vaginalis_smMIPFpraustnizii      CCAGGGTATCTAATCCTGTTTGCTA 145
                                              *************************
```

6 SNPs on 145 nt = (145-6: 139)/145 * 100 = **95.86% similarity**

2. Anaerococcus tetradius vs. Peptostreptococcus anaerobius

ROI 1

```
CLUSTAL O(1.2.4) multiple sequence alignment


Anaerococcus_tetradius_smMIPPcopri            TGATGATTTGACGTCATCCCCACCTTCCTCCGGTTTATCACCGGCAGTATATCTAGAGTC      60
Peptostreptococcus_anaerobius_smMIPPcopri     TGATGATTTGACGTCATCCCCACCTTCCTCCAGGTTATCCCTGGCAGTCTCTCTAGAGTG      60
                                              ******************************* * ***** * ****** * ******** 

Anaerococcus_tetradius_smMIPPcopri            CCCAACTTAATGATGGTAACTAAATATAGGGGTTGCGCTCGTTATGGGACTTAACCCAAC      120
Peptostreptococcus_anaerobius_smMIPPcopri     CCCAACTGAATGCTGGCAACTAAAGACAAGGGTTGCGCTCGTTGCGGGACTTAACCCAAC      120
                                              ******* **** *** ******* * * **************  ***************

Anaerococcus_tetradius_smMIPPcopri            ATCTCACGACACGAGCTGACGACAACCA     148
Peptostreptococcus_anaerobius_smMIPPcopri     ATCTCACGACACGAGCTGACGACAACCA     148
                                              ****************************
```

14 SNPs on 148 nt = (148-14: 134)/148 * 100 = **90.54% similarity**

ROI 2

```
CLUSTAL O(1.2.4) multiple sequence alignment


Anaerococcus_tetradius_smMIPCacnes           GACGACAACCATGCACCACCTGTATTACAGTCTTCCGAAGAAGTAAGGAACTATCTCTAA     60
Peptostreptococcus_anaerobius_smMIPCacnes    GACGACAACCATGCACCACCTGTCACCTCAGC----CCCGAAGGGAAGGTGTGATTAAAC     56
                                             ***********************        *       ****  * *   *   *  *

Anaerococcus_tetradius_smMIPCacnes           TTCCCTCCGTAATATGTCAAGACTTGGTAAGGTTCTTCGCGTTGCGTCGAATTAAACCAC     120
Peptostreptococcus_anaerobius_smMIPCacnes    ACCGGTCTAAGGGATGTCAAGCTTAGGTAAGGTTCTTCGCGTTGCTTCGAATTAAACCAC     116
                                               *  **      ********  * ******************** **************

Anaerococcus_tetradius_smMIPCacnes           ATGCTCCGCTGCTTGTGCGGGTCCCCGTCAATT        153
Peptostreptococcus_anaerobius_smMIPCacnes    ATGCTCCGCTACTTGTGCGGGTCCCCGTCAATT        149
                                             ********** **********************
```

42 SNPs on 149 nt = (149-42: 107)/149 * 100 = **71.81% similarity**

ROI 3

```
CLUSTAL O(1.2.4) multiple sequence alignment


Anaerococcus_tetradius_smMIPFpraustinizii          CGTCAATTCCTTTGAGTTTCACACTTGCGTGCGTACTCCCCAGGCGGAGTGTTTATTGCG     60
Peptostreptococcus_anaerobius_smMIPFpraustinizii   CGTCAATTCCTTTGAGTTTCACACTTGCGTGCGTACTCCCCAGGCGGAGTACTTAATGCG     60
                                                   **************************************************  *** ****

Anaerococcus_tetradius_smMIPFpraustinizii          TTAGCTGCGGCACCCAGAT----TATTCCAGACACCTAACACTCATCGTTTACGGCGTGG     116
Peptostreptococcus_anaerobius_smMIPFpraustinizii   TTAGCTGCGGCACCGAGGGGGGTAACCCCCGACACCTAGTACTCATCGTTTACAGCGTGG     120
                                                   ************** **       *  ** ********  ************* ******

Anaerococcus_tetradius_smMIPFpraustinizii          ACTACCAGGGTATCTAATCCTGTTTGCTA     145
Peptostreptococcus_anaerobius_smMIPFpraustinizii   ACTACCAGGGTATCTAATCCTGTTTGCTA     149
                                                   *****************************
```

17 SNPs on 145 nt = (145-17: 128)/145 * 100 = **88.28% similarity**

ROI 4

```
CLUSTAL O(1.2.4) multiple sequence alignment


Anaerococcus_tetradius_smMIPBangulatum          TTCCGATACGGCTACCTTGTTACGACTTCACCCCAGTTACTGACCCTACCTTCGACTGCT 60
Peptostreptococcus_anaerobius_smMIPBangulatum   TTCCGATACGGCTACCTTGTTACGACTTCACCCCAGTTATCGACGCCACCTTCGACGACT 60
                                                ***************************************  *** * *********  **

Anaerococcus_tetradius_smMIPBangulatum          GCTTCCTAATGGTTAGCTCACAGGCTTCGGGTATTGCCAACTTCCATGGTGTGACGGGCG 120
Peptostreptococcus_anaerobius_smMIPBangulatum   TCCTCCTTGCGGTTGGATAATCGGCTTCGGGTGTTTCCGACTCCCATGGTGTGACGGGCG 120
                                                 * ****   **** * * *  ********** ** ** *** *****************

Anaerococcus_tetradius_smMIPBangulatum          GTGTGTACAAGACCCGGGAACGCATT   146
Peptostreptococcus_anaerobius_smMIPBangulatum   GTGTGTACAAGACCCGGGAACGCATT   146
                                                **************************
```

20 SNPs on 146 nt = (146-20: 126)/146 * 100 = **86.30% similarity**

3. Peptostreptococcus anaerobius vs Gardnerella vaginalis

ROI 1

```
CLUSTAL O(1.2.4) multiple sequence alignment


Gardnerella_vaginalis_smMIPPcopri            TGATGATCTGACGTCATCCCCACCTTCCTCCGAGTTAACCCCGGCGGTCCCCCGTGAGTT    60
Peptostreptococcus_anaerobius_smMIPPcopri    TGATGATTTGACGTCATCCCCACCTTCCTCCAGGTTATCCCTGGCAGTCTCTCTAGAGTG    60
                                             ******* ***********************  **** *** *** *** * *  ****

Gardnerella_vaginalis_smMIPPcopri            CCCGGCATAACCCGCTGGCAACACAGGGCGAGGGTTGCGCTCGTTGCGGGACTTAACCCA    120
Peptostreptococcus_anaerobius_smMIPPcopri    CCCAAC--TGAATGCTGGCAACTAAAGACAAGGGTTGCGCTCGTTGCGGGACTTAACCCA    118
                                             ***  *       *********  * * * ******************************

Gardnerella_vaginalis_smMIPPcopri            ACATCTCACGACACGAGCTGACGACGACCA    150
Peptostreptococcus_anaerobius_smMIPPcopri    ACATCTCACGACACGAGCTGACGACAACCA    148
                                             ************************* ****
```

25 SNPs on 148 nt = (148-25: 123)/148 * 100 = **83.10% similarity**

ROI 2

```
CLUSTAL O(1.2.4) multiple sequence alignment


Gardnerella_vaginalis_smMIPCacnes            ---------AATTGACGGGGGCCCGCACAAGCGGCGGAG----CATGCGGATTAATTCGA    47
Peptostreptococcus_anaerobius_smMIPCacnes    GACGACAACCATGCACCACCTGTCACCTCAGCCCCGAAGGGAAGGTGTGATTAAACACCG    60
                                                       **  **       * *   ***  ** **      ** *  * **  *

Gardnerella_vaginalis_smMIPCacnes            TGC--AACGCGAAGAACCTTACCTGGGCTTGACATGTGCCTGTCGACTGCAGAGATGTGG    105
Peptostreptococcus_anaerobius_smMIPCacnes    GTCTAAGGGATGTCAAGCTTAGGTAAGGTTCTTCGCGTTGCTTCGAATTAAACCACATGC    120
                                               *  *  *     ** ****  *  * **            **** *  *   *  **

Gardnerella_vaginalis_smMIPCacnes            TTTCCCTTCGGGGCAGGTTCACAGGTGGTGCATGGTCGTCGTC    148
Peptostreptococcus_anaerobius_smMIPCacnes    TCCGCTACTTGTGCGGGTCCCCGTCAATT--------------    149
                                             *   *     * ** *** * *      *
```

95 SNPs on 148 nt = (148-95: 53)/148 * 100 = **35.81% similarity**

4. Gardnerella vaginalis vs Prevotella buccalis

ROI 1

```
CLUSTAL O(1.2.4) multiple sequence alignment


Gardnerella_vaginalis_smMIPPcopri    -TGATGATCTGACGTCATCCCCACCTTCCTCCGAGTTA------ACCCCGGCGGTCCC--    51
Prevotella_buccalis_smMIPPcopri      TGGTTGTCGTCAGCTCGTGCCGTGAGGTGTCGGCTTAAGTGCCATAACGAGCGCAACCCC    60
                                       * **   * *  ** * **        ** *  * *         *  ***   **

Gardnerella_vaginalis_smMIPPcopri    --CCGTGAGTTCCCGGCATAACCCGCTGGCAACACAGGGCGAGGGTTGCGCTCGTTGCGG    109
Prevotella_buccalis_smMIPPcopri      TTTTTTCAGTTGCCATCAGGTAATGCTGGGCACTCTGGA-------------------GA    101
                                          * **** **  **      *****  ** * **                    *

Gardnerella_vaginalis_smMIPPcopri    GACTTAACC--CAACATCTCACGACACGAGCTGACGACGACCA-------    150
Prevotella_buccalis_smMIPPcopri      TACTGCCACCGCAAGGTGTGA-GGAAGGTGGGGATGACGTCAAATCAGCA    150
                                      ***    *  ***  * * * *  * * *  ** **** * *
```

87 SNPs on 150 nt = (150-87: 63)/150 * 100 = **42.00% similarity**

ROI 2

```
CLUSTAL O(1.2.4) multiple sequence alignment


Gardnerella_vaginalis_smMIPBangulatum      AATGCGTTCCCGGGCCTTGTACACACCGCCCGTCAAGTCATGAAAGTGGGCAGCACCCGA 60
Prevotella_buccalis_smMIPBangulatum        AATACGTTCCCGGGCCTTGTACACACCGCCCGTCAAGCCATGAAAGCCGGGGGTGCCTGA 60
                                           *** ********************************* ********  **  *  ** **

Gardnerella_vaginalis_smMIPBangulatum      AGCCGGTGGCCTAACCCTTTTGGGATGGAGCCGTCTAAGGTGAGGCTCGTGATTGGGACT 120
Prevotella_buccalis_smMIPBangulatum        AGTCCGTAACC----------GTCAAGGAGCGGCCTAGGGCAAAACTGGTGATTGGGGCT 110
                                           ** * **  **          *  * ***** * *** **  *  ** ********* **

Gardnerella_vaginalis_smMIPBangulatum      AAGTCGTAACAAGGTAGCCGTACCGGAA 148
Prevotella_buccalis_smMIPBangulatum        AAGTCGTAACAAGGTAGCCGTACCGGAA 138
                                           ****************************
```

35 SNPs on 138 nt = (138-35: 103)/138 * 100 = **74.63% similarity**

In silico analyses II

1. Comparison of V3-V4 variable regions

```
CLUSTAL O(1.2.4) multiple sequence alignment


Bifidobacterium_longum      TGGGGAATATTGCACAATGGGCGCAAGCCTGATGCAGCGACGCCGCGTGAGGGATGGAGG        60
Gardnerella_vaginalis       TGGGGAATATTGCGCAATGGGGGAAACCCTGACGCAGCGACGNCGCGTGCGGGATGAAGG        60
                            ************* ******* * ** ***** ********* ****** ****** ***

Bifidobacterium_longum      CCTTCGGGTTGTAAACCTCTTTTATCGGGGAGCAAGC---GTGAGTGAGTTTACCCGTTG        117
Gardnerella_vaginalis       CCTTCGGGTTGTAAACCGCTTTTGATTGGGAGCAAGCCTTTTGGGTGAGTGTACCTTTCG        120
                            ***************** *****    **********    ** ****** ****  * *

Bifidobacterium_longum      AATAAGCACCGGCTAACTACGTGCCAGCAGCCGCGGTAATACGTAGGGTGCAAGCGTTAT        177
Gardnerella_vaginalis       AATAAGCGCCGGCTAACTACGTGCCAGCAGCCGCGGTAATACGTAGGGCGCAAGCGTTAT        180
                            ******* **************************************** ***********

Bifidobacterium_longum      CCGGAATTATTGGGCGTAAAGGGCTCGTAGGCGGTTCGTCGCGTCCGGTGTGAAAGTCCA        237
Gardnerella_vaginalis       CCGGAATTATTGGGCGTAAAGAGCTTGTAGGCGGTTCGTCGCGTCTGGTGTGAAAGCCCA        240
                            ********************* *** ******************* ********** ***

Bifidobacterium_longum      TCGCTTAACGGTGGATCCGCGCCGGGTACGGGCGGGCTTGAGTGCGGTAGGGGAGACTGG        297
Gardnerella_vaginalis       TCGCTTAACGGTGGGNTTGCGCCGGGTACGGGCGGGCTAGAGTGCAGTAGGGGAGACTGG        300
                            **************    ******************** ****** **************

Bifidobacterium_longum      AATTCCCGGTGTAACGGTGGAATGTGTAGATATCGGAAGAACACCAATGGCGAAGGCAG        357
Gardnerella_vaginalis       AATTCTCGGTGTAACGGTGGAATGTGTAGATATCGGAAGAACACCAATGGCGAAGGCAG        360
                            ***** ******************************************************

Bifidobacterium_longum      GTCTCTGGGCCGTTACTGACGCTGAGGAGCGAAAGCGTGGGGAGCGAACA  407
Gardnerella_vaginalis       GTCTCTGGGCTGTTACTGACGCTGAGAAGCGAAAGCGTGGGGAGCGAACA  410
                            ********** *************** ***********************
```

37 SNPs on 410 nt = (410-37: 373)/410 * 100 = **90.97% similarity**

2. Comparison of the five ROIs from V5 – V9 variable regions

ROI from VR V7

```
CLUSTAL O(1.2.4) multiple sequence alignment


Bifidobacterium_longum_ROI_VR7_Prevotella_copri_NZ_GG703855_1_rna43_0029      TGATGATCTGACGTCATCCCCACCTTCCTCCGAGTTAACCCCGGCGGTCCCCCGTGAGTT      60
Gardnerella_vaginalis_ROI_VR7_Prevotella_copri_NZ_GG703855_1_rna43_0029       TGATGATCTGACGTCATCCCCACCTTCCTCCGAGTTAACCCCGGCGGTCCCCCGTGAGTT      60
                                                                              ************************************************************

Bifidobacterium_longum_ROI_VR7_Prevotella_copri_NZ_GG703855_1_rna43_0029      CCCGGCATAATCCGCTGGCAACACGGGGCGAGGGTTGCGCTCGTTGCGGGACTTAACCCA      120
Gardnerella_vaginalis_ROI_VR7_Prevotella_copri_NZ_GG703855_1_rna43_0029       CCCGGCATAACCCGCTGGCAACACAGGGCGAGGGTTGCGCTCGTTGCGGGACTTAACCCA      120
                                                                              ********** ************* ***********************************

Bifidobacterium_longum_ROI_VR7_Prevotella_copri_NZ_GG703855_1_rna43_0029      ACATCTCACGACACGAGCTGACGACGACCA    150
Gardnerella_vaginalis_ROI_VR7_Prevotella_copri_NZ_GG703855_1_rna43_0029       ACATCTCACGACACGAGCTGACGACGACCA    150
                                                                              ******************************
```

2 SNPs on 150 nt = (150-2: 148)/150 * 100 = **98.66% similarity**

ROI from VR V8

```
CLUSTAL O(1.2.4) multiple sequence alignment

Bifidobacterium_longum_ROI_VR8_Shuttleworthia_satelles_DSM_14600_NZ_GG665866_1_rna19_0005     CTTCACGCAGTCGAGTTGCAGACTGCGATCCGAACTGAGACCGGTTTTCAGGGATCCGCT    60
Gardnerella_vaginalis_ROI_VR8_Shuttleworthia_satelles_DSM_14600_NZ_GG665866_1_rna19_0005      CTTCACGTAGTCGAGTTGCAGACTACGATCCGAACTGAGACCAGTTTTAAGGGATTAGCA    60
                                                                                              ******* **************** ***************** ***** ******  **

Bifidobacterium_longum_ROI_VR8_Shuttleworthia_satelles_DSM_14600_NZ_GG665866_1_rna19_0005     CCGCGTCGCCGCGTCGCATCCCGTTGTACCGGCCATTGTAGCATGCGTGAAGCCCTGGAC    120
Gardnerella_vaginalis_ROI_VR8_Shuttleworthia_satelles_DSM_14600_NZ_GG665866_1_rna19_0005      CCATGTCACCATGAAGCAACCCGTTGTACTGGCCATTGTAGCATGCGTGAAGCCCTGGAC    120
                                                                                              **  *** **  *  *** ********** ******************************

Bifidobacterium_longum_ROI_VR8_Shuttleworthia_satelles_DSM_14600_NZ_GG665866_1_rna19_0005     GTAAGGGGCATGATGATCTGACGTCATCCC   150
Gardnerella_vaginalis_ROI_VR8_Shuttleworthia_satelles_DSM_14600_NZ_GG665866_1_rna19_0005      GTAAGGGGCATGATGATCTGACGTCATCCC   150
                                                                                              ******************************
```

15 SNPs on 150 nt = (150-15: 135)/150 * 100 = **90.0% similarity**

ROI from VR V5

```
CLUSTAL O(1.2.4) multiple sequence alignment

Bifidobacterium_longum_ROI_VR5_Mageeibacillus_indolicus_NC_013895_2_rna7_0011     AGTTTTAGCCTTGCGGCCGTACTCCCCAGGCGGGATGCTTAACGCGTTAGCTCCGACACG 60
Gardnerella_vaginalis_ROI_VR5_Mageeibacillus_indolicus_NC_013895_2_rna7_0011      -GCGAAAGCGTGG---------GGAGCGAACAGGATTAGATACCCTGGTAGTCCACGCCG 50
                                                                                   *    *** * *             *   * ****     ** *      ***    **

Bifidobacterium_longum_ROI_VR5_Mageeibacillus_indolicus_NC_013895_2_rna7_0011     GAACCCGTGGAACGGGCCCCACATCCAGCATCCACCGTTTACGGCGTGGACTACCAGGGT 120
Gardnerella_vaginalis_ROI_VR5_Mageeibacillus_indolicus_NC_013895_2_rna7_0011      TAAACGGTGGACGCTGGATGTGGGGCCCATTCCACGGGTTCTGTGTCGGAGCTAACGCGT 110
                                                                                   ** * *****    *         *    ***** * **  *    ***      * **

Bifidobacterium_longum_ROI_VR5_Mageeibacillus_indolicus_NC_013895_2_rna7_0011     ATCTAATCCT---------GTTCGCTCCCCACGCTTTCGC-    151
Gardnerella_vaginalis_ROI_VR5_Mageeibacillus_indolicus_NC_013895_2_rna7_0011      TAAGCGTCCCGCCTGGGGAGTACGGCCGCAAGGCTAAAACT    151
                                                                                        ***          ** **  * * * ***    *
```

91 SNPs on 151 nt = (151-91: 59)/151 * 100 = **39.0% similarity**

ROI from VR V6

```
CLUSTAL O(1.2.4) multiple sequence alignment

Bifidobacterium_longum_ROI_VR6_Cutibacterium_acnes_J139_NZ_ADFS01000001_1_rna32_0028     ----GACGACGACCATGCACCACC-------------TGTGAACCCGCCCCGAAGGGAAG  43
Gardnerella_vaginalis_ROI_VR6_Cutibacterium_acnes_J139_NZ_ADFS01000001_1_rna32_0028      AATTGACGGGGGC-CCGCACAAGCGGCGGAGCATGCGGATTAATTCGATGCAACGCGAAG  59
                                                                                            ****  * *   **** * *               * **  **   * * * ****

Bifidobacterium_longum_ROI_VR6_Cutibacterium_acnes_J139_NZ_ADFS01000001_1_rna32_0028     CCGTATCTCTACGACCGTCGGGAACATGTCAAGCCCAGGTAAGGTTCTTCGCGTTGCATC  103
Gardnerella_vaginalis_ROI_VR6_Cutibacterium_acnes_J139_NZ_ADFS01000001_1_rna32_0028      AACCTTACCTGGGCTTGACATGTGCCTGACGACTGCAGAGATGTGGTTTCCTTTCGGGGC  119
                                                                                              *  **  *   * *  *  * ** * *   ***  * *    ***   * *   *

Bifidobacterium_longum_ROI_VR6_Cutibacterium_acnes_J139_NZ_ADFS01000001_1_rna32_0028     GAATTAATCCGCATGCTCCGCCGCTTGTGCG-GGCCCCCGTCAATT        148
Gardnerella_vaginalis_ROI_VR6_Cutibacterium_acnes_J139_NZ_ADFS01000001_1_rna32_0028      AGGTT-------------CACAGGTGGTGCATGGTCGTCGTC----        148
                                                                                            **             * * * * ****  ** *  ****
```

84 SNPs on 148 nt = (148-84: 64)/148 * 100 = **43.2% similarity**

ROI from VR V9

```
CLUSTAL O(1.2.4) multiple sequence alignment

Bifidobacterium_longum_ROI_VR9_Bifidobacterium_angulatum_JCM_7096_GCF_0010251551_ASM102515v1_NZ_AP012322_1_rna0_0019     TTCCGGTACGGCTACCTTGTTACGACTTAGTCCCAATCACGAGCCTCACCTTAGACGGCT  60
Gardnerella_vaginalis_ROI_VR9_Bifidobacterium_angulatum_JCM_7096_GCF_0010251551_ASM102515v1_NZ_AP012322_1_rna0_0019      ----------AATGCGTTCCCGGGCCTTGTACACACCGCCCGTCAAGTCATGAAAGTGGG  50
                                                                                                                                     * * **     * ***   * **    *   *    * * * *  *

Bifidobacterium_longum_ROI_VR9_Bifidobacterium_angulatum_JCM_7096_GCF_0010251551_ASM102515v1_NZ_AP012322_1_rna0_0019     CCATCCCACAAGGGGTTAGGC-CACCGGCTTCGGGTGCTGCCCACTTTCATGACTT----  115
Gardnerella_vaginalis_ROI_VR9_Bifidobacterium_angulatum_JCM_7096_GCF_0010251551_ASM102515v1_NZ_AP012322_1_rna0_0019      CAGCACCCGAAGCCGGTGGCCTAACCCTTTTGGGATGGAGCCGTCTAAGGTGAGGCTCGT  110
                                                                                                                          *    **  ***  * * * *  ***   ** ** **  ***  **    ***

Bifidobacterium_longum_ROI_VR9_Bifidobacterium_angulatum_JCM_7096_GCF_0010251551_ASM102515v1_NZ_AP012322_1_rna0_0019     GACGGGCGGT-----GTGTACAAGGCCCGGGAACGCATT      149
Gardnerella_vaginalis_ROI_VR9_Bifidobacterium_angulatum_JCM_7096_GCF_0010251551_ASM102515v1_NZ_AP012322_1_rna0_0019      GATTGGGACTAAGTCGTAACAAGGTAGCCGTACCGGAA-      148
                                                                                                                          **  **   *     **    * *   * * * ** *
```

88 SNPs on 148 nt = (148-88: 60)/148 * 100 = **40.5% similarity**
